# Religiosity, Psychological Distress, and Well-Being: Evaluating Familial Confounding With Multicohort Sibling Data

**DOI:** 10.1093/aje/kwab276

**Published:** 2021-11-16

**Authors:** Markus Jokela

**Keywords:** depressive symptoms, distress, fixed-effect regression, longitudinal study, religion, sibling

## Abstract

Several studies have associated religiosity with better mental health, but these studies have only partially addressed the problem of confounding. The present study pooled data from multiple cohort studies with siblings to examine whether associations between religiosity and mental health are confounded by familial factors (i.e., shared family background and siblings’ shared genetics). Data were collected between 1982 and 2017. Mental health was assessed with self-reported psychological distress (including depressive symptoms) and psychological well-being. Religious attendance was associated with lower psychological distress (standard-deviation difference between weekly vs. never attendance, B = −0.14, confidence interval (CI): −0.19, −0.09; *n* = 24,598 pairs), and this was attenuated by almost half in the sibling analysis (B = −0.08, CI: = −0.13, −0.04). Religious attendance was also related to higher well-being (B = 0.29, CI: = 0.14, 0.45; *n* = 3,728 pairs), and this estimate remained unchanged in sibling analysis. Results were similar for religiousness. The findings suggest that previous longitudinal studies may have overestimated the association between religiosity and psychological distress, as the sibling estimate was only one-third of the previously reported meta-analytical association (standardized correlation −0.03 vs. −0.08).

## Abbreviations

AddHealth: National Longitudinal Study of Adolescent Health
BHPS: British Household Panel Survey
CI: confidence interval
MIDUS: Midlife in the United States
NLSY-1979: National Longitudinal Survey of 1979
NLSY-1997: National Longitudinal Survey of 1997
NLSY-YA: National Longitudinal Survey of Young Adults
PSID: Panel Study of Income Dynamics
PSID-TA: Panel Study of Income Dynamics—Transition to Adulthood
SOEP: Socioeconomic Panel Survey
UKHLS: United Kingdom Household Longitudinal Study
WLS: Wisconsin Longitudinal Study

Religion is an important aspect of life for a large part of the world’s population. Religiosity may contribute to people’s psychological and social life, by offering, for example, life values and social connectedness. Dozens of studies have reported associations between various measures of religiosity and mental health (1–3). The most recent meta-analysis of 48 longitudinal studies (4) reported a protective association of *r* = −0.08 between religiosity and lower depressive symptoms. Attendance to religious services and self-reported importance of religion were slightly stronger correlates of mental health than the more private religious behaviors, such as praying and religious beliefs (4).

In determining whether religiosity actually influences mental health, the previous longitudinal studies have been valuable in addressing the issue of temporal ordering and in adjusting for many observed confounding variables (5). However, the longitudinal studies may not have taken into account all possible confounding factors, as there may be unobserved confounders that affect both religiosity and mental health, which the longitudinal study design alone may not capture adequately. Additional study designs are needed to fully assess the possible role of unobserved confounders.

The present study used sibling analysis to evaluate whether familial factors confound the associations between religiosity and mental health. Sibling comparisons can adjust for confounding due to shared family background (e.g., religious upbringing) and approximately half of any genetic confounding, as siblings share their family background and half of their alleles (6–8); sibling analysis of monozygotic twins can fully adjust for genetic confounding, as monozygotic twins share all of their alleles.

The main outcome of the current analysis was psychological distress (i.e., nonspecific symptoms of depression, anxiety, stress, and fatigue), assessed longitudinally in the next study wave following the assessment of religiosity. It has been suggested that religiosity might also be relevant for human flourishing and the more positive aspects of mental health, including personal growth, autonomy, and purpose (9, 10), so I included psychological well-being as a secondary outcome.

I also examined the dose-response associations and the possibility of reverse temporal ordering, that is, mental health predicting later religiosity. The original purpose of this study was to also use within-individual, fixed-effect panel analysis to adjust for all confounding factors that remain stable within individuals across measurement times (11, 12). However, a reviewer argued that this analysis would be invalid because the within-individual analysis assumes no reverse causation (13, 14) and that it should therefore not be included in the manuscript. Poor mental health has been shown to predict less frequent religious attendance over time (15, 16), so reverse causation is plausible. Given these uncertainties, the fixed-effect panel analysis is reported only in the online supplementary material for readers who might be interested in these results even if the assumptions of the analysis may not have been met.

## METHODS

Participants were from the National Longitudinal Study of Adolescent Health (AddHealth); British Household Panel Survey (BHPS); UK Household Longitudinal Study (UKHLS); Panel Study of Income Dynamics (PSID) and its Transition to Adulthood substudy (PSID-TA); National Longitudinal Surveys 1979 (NLSY-1979), Young Adults (NLSY-YA; who were children of the participants of NLSY-1979), and 1997 (NLSY-1997); Midlife in the United States (MIDUS); Wisconsin Longitudinal Study (WLS); German Socioeconomic Panel Survey (SOEP); and TwinLife. The cohort studies are described in more detail in Web Appendix 1 (available at https://doi.org/10.1093/aje/kwab276). The data collections of AddHealth, MIDUS, NLSY-YA, WLS, and TwinLife were specifically aimed at collecting data from siblings and/or twins. The other cohort studies were household-based studies in which sibling pairs could be identified based on common parents or if both siblings had been in the same household at least in 1 point of the data-collection period. Only full siblings and twins were included in the within-sibling analysis, and, to reduce the risk of including incorrectly identified sibling pairs, only sibling pairs with a maximum age difference of 15 years were included.

### Exposure and outcomes

Religious attendance was assessed as the frequency with which the participant reported going to church or other religious activities. The response categories were harmonized across studies as follows: 0 = never or seldom, 1 = few times a year or only special occasions, 2 = 1–2 times per month, 3 = weekly or more often. Depending on the study, religiousness was assessed with questions on how religious the participant was, how important religion was to the participant, or how much difference religious beliefs made in the participant’s life. The response scales were fairly similar across studies and were harmonized so that 0 = not religious, 1 = somewhat religious, 2 = religious, and 3 = very religious (see Web Tables 1 and 2 for details).

Psychological distress was assessed using the 12-item General Health Questionnaire in BHPS and UKHLS; the 6-item Kessler (K6) Psychological Distress Scale in MIDUS, PSID, and PSID-TA; Center for Epidemiological Studies–Depression scale in AddHealth (19 items), NLSY-79 (7 items), NLSY-YA (7 items), NLSY-97 (5 items), and WLS (20 items); a 6-item scale in SOEP (pressed for time, run-down/melancholic, well-balanced, energetic, accomplished less due to emotional problems, less careful due to emotional problems); and a 5-item scale in TwinLife (being often worried, being often unhappy/depressed, being nervous in new situations, having lot of fears, and having frequent headaches/stomach aches). I refer to these measures with the general term “psychological distress,” although some of them assessed depressive symptoms more specifically. The content overlap between measures of psychological distress and depressive symptoms was sufficiently high to be included in a single meta-analysis. For example, the Kessler Psychological Distress Scale asks the participants to rate their feelings of nervousness, hopelessness, restlessness, sadness, worthlessness, and effortfulness. Items of the Center for Epidemiological Studies–Depression scale, likewise, include questions about feelings of depression, sadness, restless sleep, happiness, loneliness, and ability to enjoy life, among others. Psychological well-being was assessed using Ryff’s Psychological Well-Being scales in MIDUS (18 items), WLS (42 items), and PSID-TA (6 items).

**Figure 1:**
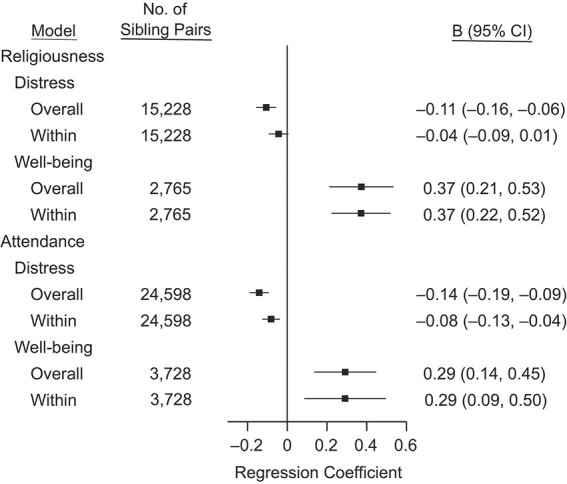
Summary estimates of random-effect meta-analyses of sibling analyses in which religiosity (religiousness and religious attendance, separate models) at wave *T* was used to predict subsequent mental health at the following study wave *T* + 1, multiple countries, 1982–2017. Regression coefficients indicate the standardized mean difference in outcome between highest and lowest values of the predictor (i.e., not vs. very religious for religiousness; never vs. weekly for attendance). “Overall” models apply ordinary regression; “within” models apply fixed-effect estimation within sibling pairs.

### Statistical analysis

Previous reviews have emphasized the importance of using longitudinal rather than cross-sectional data to assess religiosity and mental health (2, 4). Accordingly, all the main analyses were fitted using religious attendance or religiousness at time *T* to predict psychological distress or well-being at time *T* + 1 in the next study wave. TwinLife was an exception as it had only cross-sectional data, so the analysis for this cohort was based on cross-sectional measurements. Random-intercept multilevel regression was used to estimate the associations, and a fixed-effect estimator was used to test whether differences in religiosity between siblings were related to the siblings’ differences in mental health. In all analyses, each participant could contribute multiple person-observations if the participant had 2 or more repeated measurements of both religiosity and mental health; the sibling analysis was fitted with all the available repeated measurement times in which both siblings had data. In the main analysis, each sibling pair was treated separately so that families with multiple siblings could contribute several sibling pairs to the data set (e.g., 3 siblings would be included as pairs of A–B, A–C, and B–C). Standard errors were calculated using robust estimation with family as the clustering variable. In a sensitivity analysis, only 1 randomly selected sibling pair from each family was included in the analysis, so that the same individual could not be a member of more than 1 pair; this sensitivity analysis did not substantially change the results (details below). Separate models were fitted for religious attendance and religiousness; that is, they were not mutually adjusted in any of the models.

The reverse temporal ordering between religiosity and mental health was assessed by using psychological distress or well-being at wave *T* to predict religious attendance or religiousness at *T* + 1 in the next study wave. Dose-response associations were assessed by using religiousness and religious attendance as categorical variables in the multilevel regression, without the sibling comparison.

Psychological distress and psychological well-being scales were standardized within each study (standard deviation = 1). Religiousness and religious attendance scales were both divided by 3, so that their regression coefficients gave the standardized mean difference in the outcome between the lowest and highest category of religiousness (i.e., not religious vs. very religious) and religious attendance (i.e., never vs. weekly attendance), with the 1-unit difference in religiosity now referring to the difference between highest and lowest value of religiosity (range 0 to 1). To facilitate the comparison with earlier meta-analyses, the summary estimates were also calculated with standardized regression coefficients, that is, by standardizing both the measures of mental health and religiosity before running the regression analysis so as to obtain correlations that have been used in previous studies.

**Figure 2:**
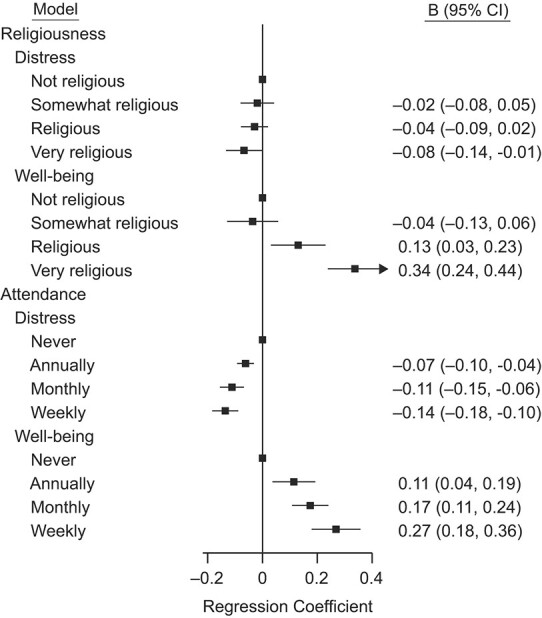
Summary estimates of random-effect meta-analyses for dose-response analyses of religiosity (religiousness and religious attendance, separate models) and subsequent mental health assessed in the following study wave *T* + 1, multiple countries, 1982–2017. Regression coefficients are from ordinary regression models and indicate the standardized mean difference in comparison with the reference group (“not religious” for religiousness and “never” for attendance).

## RESULTS

Web Table 3 shows the descriptive statistics of the samples. Web Table 4 shows the study years in which data were collected for each cohort study, and Web Table 5 shows the intraclass correlations of the variables within sibling pairs. Figure 1 shows the meta-analytical results of the sibling analyses. Religiousness and religious attendance were associated with lower psychological distress. When applying the within-pair estimation, these associations were attenuated by about half (from B = −0.14 to B = −0.08 standard-deviation difference, for weekly vs. never religious attendance; from B = −0.11 to B = −0.04 standard-deviation difference for very vs. not religious) but did not disappear completely. Associations with psychological well-being were stronger than those with distress (B = 0.29 for religious attendance, B = 0.37 for religiousness), and they remained almost unchanged in the within-pair analysis. Fixed-effect regressions that included only monozygotic twins (from AddHealth, MIDUS, and TwinLife) had confidence intervals (CIs) too wide to provide robust information (Web Figure 1). Regression models that included only 1 sibling pair per household in the analysis produced essentially the same results as the main analysis with multiple sibling pairs from the same household (Web Figure 2).

For the purpose of comparison with previous meta-analyses, Web Figure 3 reports the results of Figure 1 as standardized beta coefficients (i.e., standard deviation difference in the outcome associated with 1 standard-deviation difference in religiosity). For the association between religious attendance and psychological distress, the standardized coefficient was β = −0.05 (CI: −0.07, −0.03) in ordinary regression and β = −0.03 (CI: −0.05, −0.01) in sibling analysis. For religiousness, the standardized coefficient was β = −0.04 (CI: −0.05, −0.02) in ordinary regression and β = −0.02 (CI: −0.03, 0.00) in sibling analysis. The standardized coefficients were between β = 0.10 and β = 0.12 when predicting psychological well-being.

**Figure 3:**
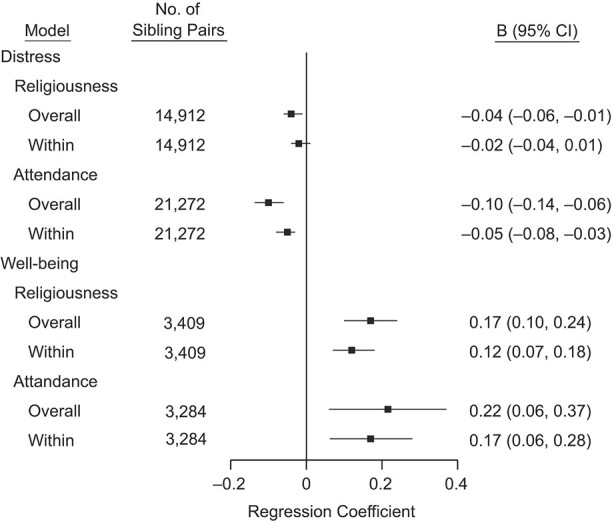
Summary estimates of random-effect meta-analyses of reverse temporal order in the sibling analysis, that is, mental health predicting later religiosity assessed in the following study wave (*T* + 1), multiple countries, 1982–2017. Regression coefficients indicate the difference in outcome associated with 1 standard deviation difference in the predictor. Overall model estimates are for analysis that does not apply fixed-effect estimation within sibling pairs, within model estimates apply fixed-effect estimation.

The dose-response associations (Figure 2) showed monotonic associations between religious attendance and psychological distress and well-being, and between religiousness and psychological well-being. In the analysis of reverse temporal order (i.e., psychological distress and well-being predicting subsequent religiousness and religious attendance), psychological distress predicted lower religiousness and less frequent religious attendance, and psychological well-being predicted higher religiousness and more frequent religious attendance (Figure 3**)**.

To illustrate the magnitude of the associations at the population level, we can consider a scenario in the United States where religious attendance has decreased from the early 1970s (11% attends never, 35% annually, 16% monthly, and 38% weekly) to the late 2010s (27% attends never, 29% annually, 15% monthly, and 28% weekly) (17, 18). If we assume that 15.0% of the population in the 1970s had at least moderate psychological distress (6-item Kessler score > 5), and use the coefficients of the sibling analysis, the population prevalence of psychological distress would have increased by 0.2 percentage points to 15.2% due to the decreasing religious attendance (see Web Table 6 for details). The same decrease in religious attendance would have decreased the average population psychological well-being from 0.000 to −0.035 (assuming baseline mean of 0 and standard deviation of 1).

Web Figures 4–11 show the study-specific associations behind the pooled associations shown in Figure 1, and the corresponding cross-sectional associations. I also fitted subgroup analyses by 1) age category (in years: <25, 25–59, ≥60), 2) region of origin (United States vs. Europe), and 3) follow-up time between study waves (in years: 1, 2, ≥3), reported in Web Tables 7 and 8. Of the 12 fixed-effect subgroup analyses, the heterogeneity was statistically significant only for one: follow-up time for religious attendance in predicting psychological well-being (stronger association in studies with 2-year interval, B = 0.57, compared to longer intervals, B = 0.21; there were no studies of well-being with 1-year intervals). However, this subgroup comparison was based on only 3 cohort studies: PSID-TA (2-year interval) versus MIDUS and WLS (longer interval).

None of the associations between religiosity and distress or well-being were observed in the within-individual analysis, in which the fixed-effect estimator attenuated the associations close to zero (reported in Web Appendix 2, Web Tables 9–11, and Web Figures 12–24).

## DISCUSSION

The present study applied sibling analysis to examine whether the associations between religiosity and mental health were confounded by familial factors, that is, shared family background and partly shared genetics of the siblings. Compared with ordinary regression, the within-sibling analysis attenuated the associations between religiosity and distress by about half, leaving an association with a standardized beta coefficient (i.e., correlation) of β = −0.03 for religious attendance and β = −0.02 for religiousness. By contrast, the sibling estimates for psychological well-being were very similar to those with ordinary regression (standardized β = 0.11 and β = 0.12). Analyses of reverse temporal order showed that psychological distress and well-being were associated with later religiosity, suggesting that both directions of temporal order were plausible.

Some limitations need to be considered. First, the self-reported measures of religiosity may be biased by misclassification due to socially desirable reporting (19). For example, objective measures of church attendance in the United States have suggested that the actual percentage of weekly church attendance may be only half of what people report in surveys (20). Second, people’s religious beliefs and behaviors may be differently motivated (e.g., fear of vengeful god vs. love of merciful god), which may partly determine the mental health correlates of religiosity, but the current measures did not query people’s religious beliefs in detail (21, 22). The analysis also did not include other possible moderator variables, such as stressful life events (23). However, religious attendance and self-reported religiousness are common measures of religiosity, so it is important to quantify their associations with mental health in the general population.

Third, the within-pair regressions are based on more limited variance in the predictor variables, which often leads to imprecise estimates. The large sample size of the present study helped to mitigate this problem. Unfortunately, the analysis of monozygotic twins was the exception here, as the CIs of these estimates were too wide to provide evidence one way or the other. Fourth, while the cohort studies of AddHealth, MIDUS, NLSY-YA, WLS, and TwinLife were specifically intended to collect sibling data, the sibling-pair data in the other 5 samples were derived from siblings who had both been in the same household in at least 1 data collection wave. Fifth, the current analysis was concerned only with psychological distress and psychological well-being, and some of the cohorts had only short or nonstandardized measures of distress. The conclusions might be different for other outcomes relevant for mental health, such as alcohol abuse or suicide (24–26). There was also variability in the assessment of religiousness and religious attendance between cohort studies, which may have introduced heterogeneity in the estimates.

The most recent meta-analysis of published studies reported an estimate of *r* = −0.08 between religiosity and mental health (4). In the present study, the correlation coefficient between religious attendance and later psychological distress in ordinary regression was β = −0.05, which was one-third weaker than the previous meta-analytical estimate. The coefficient of β = −0.03 in the sibling analysis, in turn, was two-thirds weaker than the previously estimated meta-analytical association. Thus, previous data may have overestimated the strength of the association between religiosity and psychological distress, possibly due to publication bias or incomplete assessment of confounding factors. The magnitude of the association can also be illustrated at the population level: If the sibling analysis estimated the true association between religious attendance and psychological distress, the decrease in religious attendance in the United States between the early 1970s and late 2010s would have increased the prevalence of moderate psychological distress by 0.2 percentage points (from 15.0% to 15.2%). This calculation did not take into account the possible contextual modification of the associations in which the psychological benefits of religiosity may be weaker in less religious societies (27).

It must be emphasized that the sibling analysis does not yet provide evidence for a causal association between religiosity and mental health, because it adjusts only for familial confounding factors (6). Additional quasi-experimental study designs are needed to test whether other confounding factors might bias the associations between religiosity and psychological distress. For instance, a recent Mendelian randomization study reported that belonging to a religious group was not associated with depressive symptoms in the genetic instrumental-variable regression (28).

Religiousness and religious attendance were more strongly associated with psychological well-being than with psychological distress. Psychological well-being is characterized by factors such as having autonomy and purpose in life, having opportunities for personal growth, and having meaningful relationships with other people (9). The present findings suggest that religiosity may be more important for measures of human flourishing than for measures of psychological distress. It remains to be determined whether these associations are specific to religious practices or whether they represent more general factors that also characterize secular practices (29).

In conclusion, this multicohort analysis of siblings suggested that familial confounding may account for some of the association between religiosity and psychological distress; the sibling estimate was only one-third of the previously reported meta-analytic estimate (standardized correlations of β = −0.03 vs. β = −0.08). Religiosity may be more relevant for psychological well-being than for psychological distress.

## Supplementary Material

Web_Material_kwab276
