# Correction to: Religiosity, psychological distress, and well-being: evaluating familial confounding with multicohort sibling data

**DOI:** 10.1093/aje/kwaf156

**Published:** 2026-03-25

This is a correction to: Markus Jokela, Religiosity, Psychological Distress, and Well-Being: Evaluating Familial Confounding With Multicohort Sibling Data, *American Journal of Epidemiology*, Volume 191, Issue 4, April 2022 Pages 584–590, 10.1093/aje/kwab276

In the originally published version of this manuscript, the statement of grant 345186 from the Academy of Finland was inadvertently omitted from the Acknowledgements section.

The Acknowledgements section should read:

“This work was supported by the Academy of Finland (grants 311578 and 345186).”

These details have been corrected only in this correction notice to preserve the published version of record.

