# Supplementary material for: Religiosity, Psychological Distress, and Well-Being: Evaluating Familial Confounding With Multicohort Sibling Data
Source: Am J Epidemiol. 2021 Nov 16;191(4):584–90. doi: 10.1093/aje/kwab276 (PMC8971076; doi:10.1093/aje/kwab276)
Supplement: Web_Material_kwab276 [file web_material_kwab276.pdf]

Web Material

Religiosity, Psychological Distress, and Well-Being: Evaluating Familial Confounding With Multicohort Sibling Data

Contents

Web Appendix 1 2

Web Tables 1–8 11

Web Figures 1–11 23

Web Appendix 2 35

Web Tables 9–11 37

Web Figures 12–24 40

Web Appendix 1

National Longitudinal Study of Adolescent Health (AddHealth)

The National Longitudinal Study of Adolescent Health (Add Health) is a longitudinal study of a nationally representative sample of adolescents in grades 7-12 in the United States during the 1994-1995 school year. The Add Health cohort has been followed into young adulthood with five in-home interviews. Add Health combines longitudinal survey data on respondents' social, economic, psychological and physical well-being with contextual data on the family, neighborhood, community, school, friendships, peer groups, and romantic relationships, providing unique opportunities to study how social environments and behaviors in adolescence are linked to health and achievement outcomes in young adulthood.

Study website:
https://addhealth.cpc.unc.edu

Acknowledgement: This research uses data from Add Health, a program project directed by Kathleen Mullan Harris and designed by J. Richard Udry, Peter S. Bearman, and Kathleen Mullan Harris at the University of North Carolina at Chapel Hill, and funded by grant P01-HD31921 from the Eunice Kennedy Shriver National Institute of Child Health and Human Development, with cooperative funding from 23 other federal agencies and foundations. Information on how to obtain the Add Health data files is available on the Add Health website (https://addhealth.cpc.unc.edu/). No direct support was received from grant P01-HD31921 for this analysis.

British Household Panel Survey (BHPS) and the UK Household Longitudinal Survey (UKHLS)

The British Household Panel Survey (BHPS) is a longitudinal survey of a nationally representative sample of over 5000 British households with annual follow-ups. The original cohort included 10,264 individuals aged 16-97 at baseline in 1991, and was based on a clustered, stratified sample of addresses throughout Great Britain south of the Caledonian Canal (excluding North of Scotland and Northern Ireland). New participants have been included in the sample over the years if they are born to original sample member, if they have moved into a household in the original sample, or if a member of the original sample moves into a new household with one or more new people. In addition, the sample was enriched with additional recruitment of participants at waves 9 and 11, from Scotland and Wales, and from Northern Ireland, respectively, so extending the sample to cover the whole UK. The most recent (18th) follow-up of the BHPS was carried out in 2008-2009.

After 2008-2009, the BHPS became merged with the larger UK Household Longitudinal Survey (UKHLS), also known as the Understanding Society study. The original sample for the UKHLS were from approximately 40,000 household in the United Kingdom, including participants from the BHPS, interviewed in 2009–2011. The overall purpose of Understanding Society is to provide high quality longitudinal data about subjects such as health, work, education, income, family, and social life to help understand the long- term effects of social and economic change, as well as policy interventions designed to impact upon the general well-being of the UK population.

Study websites:
http://www.understandingsociety.org.uk/
http://www.esds.ac.uk/longitudinal/access/bhps/L33196.asp

Acknowledgements: Understanding Society is an initiative funded by the Economic and Social Research Council and various Government Departments, with scientific leadership by the Institute for Social and Economic Research, University of Essex, and survey delivery by NatCen Social Research and Kantar Public. The research data are distributed by the UK Data Service. Neither the original collectors of the data nor the distributors of the data bear any responsibility for the analyses or interpretations presented here.

Data references: University of Essex. Institute for Social and Economic Research, NatCen Social Research and Kantar Public, [producers]: Understanding Society: Waves 1-6, 2009-2015 [computer file]. 8th Edition. Colchester, Essex: UK Data Service [distributor], November 2016. SN: 6614

University of Essex. Institute for Social and Economic Research. (2010). British Household Panel Survey: Waves 1-18, 1991-2009. [data collection]. 7th Edition. UK Data Service. SN: 5151, http://doi.org/10.5255/UKDA-SN-5151-1

Health and Retirement Study (HRS)

The HRS is a nationally representative longitudinal study of more than 30,000 individuals representing the U.S. population older than 50 years. Telephone or in-person interviews are conducted every 2 years, administered under the NIA and the University of Michigan's Institute for Social Research. As of 1998, the HRS consists of 4 sources of data collection: (1) The original HRS began as two distinct surveys that were merged in 1998. The original HRS was initially administered in 1992 to a nationally representative sample of Americans born in the years 1931 through 1941. In the case of married couples, both spouses (including spouses who were younger than 51 or older than 61) were also interviewed; (2) The second survey, originally referred to as the Study of Assets and Health Dynamics Among the Oldest Old (AHEAD), was first administered in 1993 to a nationally representative sample of Americans born in 1923 or earlier (n=8,000) and merged with the HRS in 1998. In the case of married couples, interviews were conducted with both spouses; (3) In 1998, a subsample of individuals born between 1924 and 1930, referred to as Children of the Depression Age (CODA) was added to HRS; (4) Another subsample consisting of people born between 1942 and 1947 (War Baby cohort) was added to replenish the sample of people in their early 50s as the original HRS cohort aged. The Health Sciences Institutional Review Board at the University of Michigan approved the HRS.

The HRS was not used in the sibling analysis but only in the Web analysis of within-individual changes.

Study website: http://hrsonline.isr.umich.edu

Acknowledgements: The HRS (Health and Retirement Study) is sponsored by the National Institute on Aging (grant number NIA U01AG009740) and is conducted by the University of Michigan. The Health and Retirement Study public use dataset (Ann Arbor, MI, 2016) is produced and distributed by the University of Michigan.

Midlife Development in the United States (MIDUS)

The MacArthur Foundation Survey of Midlife Development in the United States (MIDUS) is based on a nationally representative random-digit-dial sample of non-institutionalized, English-speaking adults, aged 25 to 74 years, selected from working telephone banks in the coterminous United States in 1995-1996. The total original sample (n=7108) includes main respondents (n=3487), their siblings (n=950), a city oversample (n=757), and a twin subsample (n=1914). Data were collected in a telephone interview and with a mail questionnaire. A follow-up study of the original cohort was conducted in 2004-2005 and 2013-2014.

Study website: http://www.midus.wisc.edu/

Acknowledgement: The MIDUS study has been funded by John D. and Catherine T. MacArthur Foundation Research Network, National Institute on Aging (P01-AG020166), and National institute on Aging (U19-AG051426)

Data references: Brim, Orville G., et al. NATIONAL SURVEY OF MIDLIFE DEVELOPMENT IN THE UNITED STATES (MIDUS), 1995-1996 [Computer file]. ICPSR02760-v4. Ann Arbor, MI: DataStat, Inc./Boston, MA: Harvard Medical School, Dept. of Health Care Policy [producers], 2007. Ann Arbor, MI: Inter-university Consortium for Political and Social Research [distributor], 2007-04-16.

National Longitudinal Survey of Youth

NLSY79: The NLSY79 is a nationally representative sample of 12,686 young men and women born during the years 1957 through 1964 and living in the United States when the survey began. The survey respondents were ages 14 to 22 when first interviewed in 1979. The cohort began with oversamples of Hispanics, economically disadvantaged nonblacks and non-Hispanics, and youths in the military. The military oversample was discontinued after the 1984 survey, and the economically disadvantaged nonblacks and non-Hispanics oversample was discontinued in 1990. Interviews were conducted annually from 1979 to 1994 and on a biennial basis thereafter. NLSY-YA: In 1986, a separate survey of all children born to NLSY79 female respondents began. The child survey includes assessments of each child as well as additional demographic and development information collected from either the mother or child. For children aged 10 and older, information has been collected from the children biennially since 1988 on a variety of factors including child-parent interaction, attitudes toward schooling, dating and friendship patterns, religious attendance, health, substance use, and home responsibilities. Biennially (since 1994), children ages 15 and older complete interviews modeled on the NLSY79 questionnaire. Information collected includes their schooling, training, work experiences and expectations, health, dating, fertility and marital histories, and household composition. Starting in 2016, NLSY79 children age 12 and older were included in the Young Adult data collection. NLSY97: The NLSY97 consists of a nationally representative sample of 8,984 men and women born during the years 1980 through 1984 and living in the United States at the time of the initial survey in 1997. Participants were ages 12 to 16 as of December 31, 1996. Interviews were conducted annually from 1997 to 2011 and biennially since then. The NLSY97 collects information on respondents' labor market behavior and educational experiences. The survey also includes data on the youths' family and community backgrounds to help researchers assess the impact of schooling and other environmental factors on these labor market entrants.

Study website: https://www.bls.gov/nls/

Acknowledgements:

The NLSY79 survey is sponsored and directed by the U.S. Bureau of Labor Statistics and managed by the Center for Human Resource Research (CHRR) at The Ohio State University. Interviews are conducted by the National Opinion Research Center (NORC) at the University of Chicago. The Children of the NLSY79 survey is sponsored and directed by the U.S. Bureau of Labor Statistics and the National Institute for Child Health and Human Development. The survey is managed by the Center for Human Resource Research (CHRR) at The Ohio State University and interviews are conducted by the National Opinion Research Center (NORC) at the University of Chicago. The NLSY97 survey is sponsored and directed by the U.S. Bureau of Labor Statistics and and managed by the Center for Human Resource Research (CHRR) at The Ohio State University. Interviews are conducted by the National Opinion Research Center (NORC) at the University of Chicago.

Panel Study of Income Dynamics (PSID)

PSID: The original PSID sample of roughly 18,000 people in 5,000 households consisted of a nationally representative sample and an oversample of low- income families. The oversample was included to facilitate investigations of poverty-related issues. PSID data include economic, social, and health information collected using in-person, telephone, and computer-assisted interviewing methods. PSID families are followed regardless of where they live. The sample grows naturally as children and grandchildren from these families form their own households and are invited to join the PSID. Samples of immigrants have been added to the PSID in 1997/1999 and 2017. PSID gathers data on the family as a whole and on individuals residing within the family, emphasizing the dynamic and interactive aspects of family economics, demography, and health. PSID data were collected annually from 1968-1997 and biennially after 1997.

PSID-TA: The Child Development Supplement (CDS) collects extensive data on children and adolescents and their primary caregivers in PSID families. The first cohort of the CDS was launched in 1997 and observed children every five years across three waves from 1997 to 2007 in order to study the dynamic process of early life human and social capital acquisition. In 2014, CDS began following a new cohort of children, conducting interviews with all eligible children in PSID families born or adopted since 1997 and their primary caregivers. The study design and questionnaire content are consistent with earlier waves of the CDS to permit cross-cohort analysis of children's development. Once children from CDS reach age 18 they are followed in the Transition into Adulthood Supplement (TAS), which started in 2005 and collects data biennially. The study captures data on developmental pathways and outcomes during the transition from childhood through young adulthood. As of 2017, all young adults age 18-28 are eligible for TAS, regardless of their participation in CDS.

Study website: https://psidonline.isr.umich.edu/default.aspx

Acknowledgements: The collection of data used in this study was partly supported by the National Institutes of Health under grant number R01 HD069609 and R01 AG040213, and the National Science Foundation under award numbers SES 1157698 and 1623684.

Data reference: Panel Study of Income Dynamics, public use dataset. Produced and distributed by the Survey Research Center, Institute for Social Research, University of Michigan, Ann Arbor, MI, 2020.

Socio-Economic Panel Study (SOEP)

The German Socio-Economic Panel Study (SOEP) is a longitudinal study of private households. The study started in 1984 in West Germany with two subsamples: Sample A, the main sample, covering the population of private households, and Subsample B, which oversampled the "guest worker households" with Turkish, Spanish, Italian, Greek and Yugoslavian household heads. The original sample included 5921 households and 12,245 individual respondents. Several additional samples have subsequently been integrated in the study, including a sample of Germans from the late East Germany in 1990 (2,179 households; 4,453 individuals), an immigrant sample in 1994/1995 (522 households; 1,078 individuals), a refreshment sample of existing subsamples in 1998 (1,056 households; 1,910 individuals), an "innovation" subsample again covering all existing subsamples in 2000 (6,043 households; 10,880 individuals), a high-income subsample of households with net earnings more than 4500 euros/month in 2002 (1,224 households; 2,671 individuals), a second refreshment sample covering all existing subsamples in 2006 (1,506 households; 2,616 individuals), and an "incentive" sample covering all existing subsamples in 2009 (1,531 households; 2,509 individuals). All household members aged 17 years or older are invited for interview, which are carried out annually. Altogether, a total of 34,881 individuals have participated in the study at least in one study wave.

Study website: http://www.diw.de/en/soep

Acknowledgement: The data used in this publication were made available to us by the German Socio-Economic Panel Study (SOEP) at the German Institute for Economic Research (DIW), Berlin.

Data reference: Wagner, Gert G., Frick, Joachim R., and Schupp, Jürgen (2007), The German Socio-Economic Panel Study (SOEP) – Scope, Evolution and Enhancements, Schmollers Jahrbuch 127 (1), 139-169.

German Twin Family Panel (Twinlife)

The German Twin Family Panel, 'TwinLife', collects longitudinal data on, in the first wave, 4097 families with monozygotic or same-sex dizygotic twin children. The base population of TwinLife consists of twins aged 5, 11, 17, and 23 to 24 at the time of the first survey. The study focuses on genetic and environmental influences on social inequality with a focus on families with monozygotic or dizygotic twin children. To exclude effects of within-twin-pair gender differences, the study includes only same-sex dizygotic twins. As of 2019, two face-to-face, at-home interviews and two telephone interviews have been conducted. The planned observation period comprises 10 years (between 2014 and 2023). TwinLife is the first twin (family) panel in Germany implementing a population register-based sampling design, which allows for reliable comparisons with twin data from other countries. Additionally, TwinLife covers all parts of the country and includes the whole range of the educational, occupational and income structure.

Study website: https://www.diw.de/en/diw_01.c.432303.en/twinlife_short_description.html

Acknowledgements: The TwinLife project is funded by the German Research Foundation (DFG) (grant number 220286500) awarded to Martin Diewald, Rainer Riemann, and Frank M. Spinath.

Wisconsin Longitudinal Study (WLS), Graduate and Sibling Samples

Graduate sample. The Wisconsin Longitudinal Study has followed a random sample of 10317 participants (5326 women, 4991 men) who were born between 1937 and 1940 and who graduated from Wisconsin high schools in 1957. After baseline data collection in 1957, survey data have been collected from the participants or their parents in 1964, 1975, 1992/3, 2003/5, and 2011. The present study used data from the 1993 follow-up. The WLS sample is broadly representative of white, non-Hispanic American men and women who have completed at least a high school education (among Americans aged 50 to 54 in 1990 and 1991, approximately 66 percent were non-Hispanic white persons who completed at least 12 years of schooling). It is estimated that about 75 percent of Wisconsin youth graduated from high school in the late 1950s – everyone in the primary WLS sample graduated from high school.

Sibling sample. In addition to the main sample of the 1957 high school graduates, the WLS has also collected data on a selected sibling of a sample of the graduates. The data collection in adulthood has been very similar although not entirely identical for the siblings as for the graduates. For the present purposes, the sibling sample was analyzed separately from the graduate sample, because the sampling frame of the individuals for the graduate cohort and sibling cohort was considered to sufficiently to justify the decision of not combining the samples.

Study website: http://www.ssc.wisc.edu/wlsresearch/

Acknowledgements: The research uses data from the Wisconsin Longitudinal Study (WLS) of the University of Wisconsin-Madison. Since 1991, the WLS has been supported principally by the National Institute on Aging (AG-9775 and AG-21079), with additional support from the Vilas Estate Trust, the National Science Foundation, the Spencer Foundation, and the Graduate School of the University of Wisconsin-Madison. A public use file of data from the Wisconsin Longitudinal Study is available from the Wisconsin Longitudinal Study, University of Wisconsin-Madison, 1180 Observatory Drive, Madison, Wisconsin 53706 and at http://www.ssc.wisc.edu/wlsresearch/data/. The interpretations, opinions, and inferences based on the data are solely the responsibility of the authors.

Web Table 1. Response options and scoring of religious attendance

**AddHealth**	*Waves 1 & 2*		*Wave 3, 4, and 5*	
	In the past 12 months, how often did you attend religious services?		How often have you attended {church/ synagogue/ temple/ mosque/ religious} services last year	
	3	Once a week or more	0	Never
	2	Once a month or more/less than once a week	1	A few times
	1	Less than once a month	1	Several times
	0	Never	2	Once a month
			2	2 or 3 times a month
			3	Once a week
			3	More than once a week
**BHPS**	How often, if at all, do you attend religious services or meetings			
	3	Once a week or more		
	2	Less often but at least once a month		
	1	Less often but at least once a year		
	0	Never or practically never		
**HRS**	About how often have you attended religious services during the past year?			
	3	More than once a week		
	3	Once a week		
	2	Two or three times a month		
	1	One or more times a year		
	0	Not at all		
**MIDUS**	How often do you usually attend religious or spiritual services?			
	3	More than once a week		
	3	About once a week		
	2	One to three times a month		
	1	Less than once a month		
	0	Never		
**NLSY-79**	In the past year, about how often have you attended religious services			
	3	More than once a week		
	3	About once a week		
	2	Two or three times a month		
	2	About once a month		
	1	Several times a year or less		
	0	Not at all		
**NLSY-YA**	In the past year about how often have you attended religious services?			

3 More than once a week
3 About once a week
2 Two or three times a month
2 About once a month
1 Several times a year or less
0 Not at all

NLSY-97 In the past 12 months, how often have you attended a worship service like a church or synagogue service, or a service at a mosque?

0 Never
1 Once or twice
1 Less than once a month
2 About once a month
2 About twice a month
3 About once a week
3 Several times a week
3 Everyday

PSID † How often did you go to religious services?
– Expressed as times per year/month/week/day

PSID-TA † How often did you go to religious services?--
– Expressed as times per year/month/week/day

SOEP Attend church or other religious events

3 Every Week
2 Every Month
1 Less Frequently
0 Never

UKHLS How often, if at all, do you attend religious services or meetings

3 Once a week or more
2 Less often but at least once a month
1 Less often but at least once a year
0 Never or practically never
0 Only at weddings, funerals etc.

Twinlife Frequency of visiting house of worship

3 At least once a week
2 At least once a month
1 Several times a year
1 Once a year or less often
0 Never

**WLS †**	Frequency of religious attendance<br>– Expressed as times per year/month/week/day

Note: † The participant could choose whether to report attendance as the number of times in either year, month, week, or day, depending on the participant's frequency of religious attendance. For example, one participant could report attending 2 times per week and another could report 3 times per year (i.e., number of times and unit of time were coded as separate variables).

Web Table 2. Response options and scoring of religiousness

**AddHealth**	*Waves 1 & 2*<br>How important is religion to you?<br>3 Very important<br>2 Fairly important<br>1 Fairly unimportant<br>0 Not important at all	*Waves 3, 4, and 5*<br>How important is your religious faith to you?<br>0 Not important<br>1 Somewhat important<br>2 Very important<br>3 More important than anything else
**BHPS**	How much difference would you say religious beliefs make to your life? Would you say they make<br>1 A little difference<br>2 Some difference<br>3 A great difference?<br>0 None/no difference	
**HRS**	How important would you say religion is in your life<br>3 Very important<br>2 Somewhat important<br>0 Not too important	
**MIDUS**	How religious are you?<br>0 Not at all<br>1 Not very<br>2 Somewhat<br>3 Very	
**NLSY-YA**	How important is religion to you?<br>3 Very important<br>2 Fairly important<br>1 Fairly unimportant<br>0 Not important at all	
**NLSY-97**	How important is religious faith in daily life to you?<br>3 Extremely important<br>2 Very important<br>1 Somewhat important<br>1 Not very important<br>0 Not important at all	
**PSID-TA**	How important is religion to you?<br>0 Not at all important<br>1 Not very important<br>2 Somewhat important	

	3	Very important
**WLS**		How religious are you?
	0	1 Not at all
	1	2 Not very
	1	3 Somewhat
	2	4 Very
	3	5 Extremely

Web Table 3. Descriptive statistics of person-observations across all study waves

	AddHealth	BHPS	MIDUS	NLSY79	NLSY97	NLSY-YA
Religious attendance						
Never	3033 (25.3)	3981 (67.1)	1107 (21.6)	2264 (20.9)	11145 (30.8)	8215 (24.2)
Few times a year	2828 (23.6)	972 (16.4)	1325 (25.8)	2806 (25.9)	12466 (34.4)	8698 (25.6)
Monthly/2-3 per month	2202 (18.4)	353 (6.0)	686 (13.4)	1469 (13.5)	5004 (13.8)	7664 (22.5)
Weekly/More often	3930 (32.8)	624 (10.5)	2016 (39.3)	4305 (39.7)	7604 (21)	9421 (27.7)
Religiousness						
Not religious	1845 (15.4)	3276 (55.2)	304 (5.9)	–	1296 (3.6)	2920 (8.6)
Somewhat religious	1990 (16.6)	1195 (20.2)	1015 (19.8)	–	1563 (4.3)	2756 (8.1)
Religious	4497 (37.5)	874 (14.7)	2420 (47.1)	–	1530 (4.2)	11717 (34.5)
Very religious	3666 (30.6)	588 (9.9)	1389 (27.1)	–	1430 (3.9)	16539 (48.6)
Women	6292 (52.4)	2929 (49.4)	2993 (58.2)	5364 (49.5)	18294 (50.4)	17232 (50.6)
Age (mean, sd)	20.0 (5.4)	24.1 (8.5)	50.3 (12.2)	39.1 (2)	23.6 (4.4)	20.9 (4.1)
n(persons)	3544	2457	1811	3736	1736	5475
n(sibling pairs)	1914	1673	1302	2826	1944	4550

Web Table 3 continues….

… Web Table 3 continues

	PSID	PSID-TA	SOEP	TWINLIFE	UKHLS	WLS
Religious attendance						
Never	2894 (30.9)	572 (31.4)	9466 (53.2)	1241 (63.5)	2851 (54.6)	968 (13.7)
Few times a year	1751 (18.7)	475 (26.1)	5643 (31.7)	483 (24.7)	698 (13.4)	882 (12.5)
Monthly/2-3 per month	1955 (20.9)	415 (22.8)	1526 (8.6)	137 (7.0)	551 (10.6)	1369 (19.4)
Weekly/More often	2751 (29.4)	357 (19.6)	1165 (6.5)	93 (4.8)	1117 (21.4)	3829 (54.3)
Religiousness						
Not religious	–	397 (21.8)	–	614 (31.4)	1924 (36.9)	533 (7.6)
Somewhat religious	–	181 (10.0)	–	918 (47.0)	845 (16.2)	1152 (16.3)
Religious	–	567 (31.2)	–	369 (18.9)	945 (18.1)	1183 (16.8)
Very religious	–	677 (37.2)	–	52 (2.7)	1501 (28.8)	187 (2.7)
Women	3989 (42.7)	981 (53.8)	8304 (46.7)	1116 (57.1)	2648 (50.8)	4127 (53.8)
Age (mean, sd)	40.7 (11.0)	21.1 (2.2)	27.7 (8.3)	19.3 (3)	22.9 (9.1)	57.6 (7)
n(persons)	2789	736	4592	1942	1690	4224
n(sibling pairs)	2399	373	2694	974	1976	2112

Note: Descriptive statistics are reported for person-observations of individuals across all the study waves in which the participant and his/her sibling had data on at least one exposure variable (religious attendance and/or religiousness) and at least one outcome variable (psychological distress and/or psychological wellbeing). n(persons) indicates the number of unique individuals, and n(sibling pairs) indicates the number of unique sibling pairs. In households with more than two siblings, all possible sibling pairs were formed (e.g., three siblings would form three sibling pairs of A–B, A–C, and B–C), so the number of sibling pairs could exceed the number of persons divided by two.

Web Table 4. Study years of the cohort studies included in the analysis.

Year	AddHealth	BHPS	HRS	MIDUS	NLSY79	NLSY97	NLSY-YA	PSID	PSID-TA	SOEP	TWINLIFE	UKHLS	WLS
1982	–	–	–	–	X	–	–	–	–	–	–	–	–
1991	–	X	–	–	–	–	–	–	–	–	–	–	–
1992	–	X	–	–	–	–	–	–	–	–	–	–	–
1993	–	X	–	–	–	–	–	–	–	–	–	–	X
1994	–	X	–	–	–	–	X	–	–	–	–	–	–
1995	X	X	–	X	–	–	–	–	–	–	–	–	–
1996	X	X	–	–	–	–	X	–	–	–	–	–	–
1997	–	X	–	–	–	–	–	–	X	–	–	–	–
1998	–	X	–	–	–	–	X	–	–	–	–	–	–
1999	–	X	–	–	–	–	–	–	X	–	–	–	–
2000	–	X	–	–	X	X	X	–	–	–	–	–	–
2001	X	X	–	–	–	X	–	–	X	X	–	–	–
2002	–	X	–	–	–	X	X	–	–	X	–	–	–
2003	–	X	–	–	–	X	–	X	X	X	–	–	–
2004	–	X	X	X	–	X	X	–	–	X	–	–	–
2005	–	X	–	–	–	X	–	X	X	X	–	–	–
2006	–	X	X	–	–	X	X	–	–	X	–	–	X
2007	–	X	–	–	–	X	–	X	X	X	–	–	–
2008	X	X	X	–	–	X	X	–	–	X	–	–	–
2009	–	–	–	–	–	–	–	X	X	X	–	–	–
2010	–	–	X	–	–	X	X	–	–	X	–	X	–
2011	–	–	–	–	–	X	–	X	X	X	–	–	X
2012	–	–	X	–	–	–	X	–	–	X	–	X	–
2013	–	–	–	X	–	–	–	X	X	X	–	–	–
2014	–	–	X	–	–	–	X	–	–	X	–	–	–
2015	–	–	–	–	–	X	–	X	X	X	X	–	–
2016	–	–	X	–	–	–	X	–	–	X	–	–	–
2017	X	–	–	–	–	X	–	X	X	X	–	X	–

Note: In each study, data for a single study wave may have been collected over several adjacent years. In the table, the study wave year is determined as the rounded mean year of the data collection in order to have one year to represent one study wave. Health and Retirement Study (HRS) was not included in the sibling analysis.

Web Table 5. Intraclass correlations of the variables within sibling pairs.

	**Religious attendance**	**Religiousness**	**Psychological distress**	**Psychological wellbeing**
AddHealth	0.55	0.51	0.21	–
BHPS	0.63	0.44	0.10	–
MIDUS	0.32	0.28	0.18	0.18
NLSY79	0.31	–	–	–
NLSY97	0.39	0.40	0.15	–
NLSY-YA	0.43	0.41	0.13	–
PSID	0.28	–	0.11	–
PSID-TA	0.44	0.37	0.15	0.00
SOEP	0.57	–	0.30	–
TWINLIFE	–	–	–	–
UKHLS	0.64	0.70	0.15	–
WLS	0.21	–	0.07	0.13

Note: Values are intraclass correlations within sibling pairs in a pooled sample of all the cohort studies, indicating the proportion of total variance in the variable accounted for by average differences between sibling pairs.

Web Table 6. Illustration of how the population prevalence change was calculated for sibling analysis of religious attendance and psychological distress.

	**Early 1970s**				**Late 2010s**			
**Religious attendance**	c	p	1-p	odds	c	p	1-p	odds
Never	0.11	0.1614	0.8386	0.1925	0.27	0.1614	0.8386	0.1925
Few times a year	0.35	0.1550	0.8450	0.1834	0.29	0.1550	0.8450	0.1834
Monthly/2-3 per month	0.16	0.1487	0.8513	0.1747	0.15	0.1487	0.8513	0.1747
Weekly/More often	0.38	0.1427	0.8573	0.1665	0.28	0.1427	0.8573	0.1665
	Total	0.1500			Total†	0.1523		

Note: c = proportion of the population in the religious attendance category; p = prevalence of psychological distress in category c; odds = p/(1-p); Total = Population prevalence of distress (weighted average). † Total estimate for late 2010s is 0.1508/0.99 = 0.1523 to adjust for 1% of non-responders.

Population-level estimates: The hypothetical scenario starts with the assumption that the prevalence of moderate psychological distress in the population in the early 1970s was 15.0%, and that the frequencies of religious attendance were 11% never, 35% few times a year, 16% monthly/2-3 per month, and 38% weekly/more often, as they were in the United States. On a scale of 0 (never) to 3 (weekly), a 1-unit difference in religious attendance was associated with -0.08SD / 3 = 0.027SD difference in psychological distress in the sibling analysis, which converts to an odds ratio of OR = 0.953 with the formula: log(OR) = SMD * (π/sqrt(3)), where OR = odds ratio and SMD = standardized mean difference.

In order to have the population prevalence of distress of 15.0% in the 1970s, we set the distress prevalence for the category "never" to p_0=0.1614. The odds ($p_x/(1-p_x)$) for the other attendance categories *x* can then be calculated as $odds_x = (p_0/(1-p_0)) * 0.953^x$, from which the distress prevalence for each category can be calculated as $p_x = odds_x / (1+odds_x)$. To calculate the increase in population distress prevalence associated with declining religious attendance between early 1970s and late 2010s, we keep the distress prevalence in each category the same as it was in the 1970s scenario; only the proportions of the categories change according to General Social Survey data cited in the main manuscript (27% never, 29% few times a year, 15% monthly/2-3 per month, and 28% weekly/more often). Finally, population prevalence of distress can be calculated as the weighted average of distress prevalence of the different attendance categories. For the values of late 2010s, the weighted sum is divided by 0.99 to take into account that 1% of respondents had reported "don't know" for the question. Web Table 8 above illustrates the calculations for the sibling analysis of religious attendance and psychological distress.

Web Table 7. Meta-analytic associations of religiousness by subgroup (fixed-effect sibling analysis).

	B (95% CI)	n(pairs)
**Religiousness (t) and psychological distress (t+1)**		
Age †		
<25	-0.05 (-0.12, 0.01)	10158
25–60	-0.01 (-0.17, 0.16)	4674
60+	-0.09 (-0.37, 0.19)	1028
Geographic region		
Europe	0.01 (-0.10, 0.11)	3648
United States	-0.07 (-0.13, -0.01)	10607
Study wave interval		
1 year	0.00 (-0.10, 0.10)	3247
2 years	-0.06 (-0.14, 0.03)	4918
>3 years	-0.08 (-0.16, 0.00)	6090
**Religiousness (t) and psychological wellbeing (t+1)**		
Age †		
<25	0.43 (0.19, 0.68)	365
25–60	0.59 (0.14, 1.03)	1033
60+	0.30 (0.06, 0.53)	1120
Geographic region		
Europe ‡	–	–
United States	0.37 (0.22, 0.52)	2765
Study wave interval		
1 year ‡	–	–
2 years	0.50 (0.26, 0.74)	373
>3 years	0.31 (0.13, 0.48)	2392

Note: Values are regression coefficients (and 95% confidence intervals) of fixed-effect regressions of within-sibling-pair regressions and within-individual regressions, indicating the difference in psychological distress and psychological wellbeing in standard deviations between 0=not at all religious and 3=very religious individuals. n(pairs) = Number of sibling pairs, n(persons) = Number of persons. Study wave interval refers to the time interval between the assessment of religiosity at time T and psychological distress or wellbeing at time T+1. † Age groups were analyzed separately within each study, so the same individual could be included in more than one age category at different points of the study follow-up time. ‡ Data not available. None of the heterogeneity estimates for the subgroup analyses were statistically significant.

Web Table 8. Meta-analytic associations of religious attendance by subgroup (fixed-effect sibling regressions)

	Sibling analysis	
	B (95% CI)	n(pairs)
**Religious attendance (t) and psychological distress (t+1)**		
Age †		
<25	-0.05 (-0.14, 0.04)	13749
25–60	-0.06 (-0.12, -0.01)	12241
60+	-0.18 (-0.37, 0.01)	970
Geographic region		
Europe	0.01 (-0.19, 0.20)	6342
United States	-0.10 (-0.14, -0.07)	17282
Study wave interval		
1 year	-0.08 (-0.21, 0.05)	6310
2 years	-0.12 (-0.18, -0.06)	7315
>3 years	-0.06 (-0.13, 0.01)	9999
**Religious attendance (t) and psychological wellbeing (t+1)**		
Age †		
<25	0.50 (0.21, 0.80)	365
25–60	0.23 (-0.04, 0.49)	2625
60+	0.28 (0.10, 0.45)	1004
Geographic region		
Europe ‡	–	–
United States	0.29 (0.09, 0.50)	3728
Study wave interval		
1 year ‡	–	–
2 years	0.57 (0.29, 0.85) *	373
>3 years	0.21 (0.04, 0.37) *	3355

Note: Values are regression coefficients (and 95% confidence intervals) of fixed-effect regressions of within-sibling-pair regressions and within-individual regressions, indicating the difference in psychological distress and psychological wellbeing in standard deviations between 0=never attending religious services and 3=weekly attendance of religious services. n(pairs) = Number of sibling pairs, n(persons) = Number of persons. Study wave interval refers to the time interval between the assessment of religiosity at time T and psychological distress or wellbeing at time T+1. † Age groups were analyzed separately within each study, so the same individual could be included in more than one age category at different points of the study follow-up time. ‡ Data not available.
* Heterogeneity estimate for the subgroup analysis statistically significant.

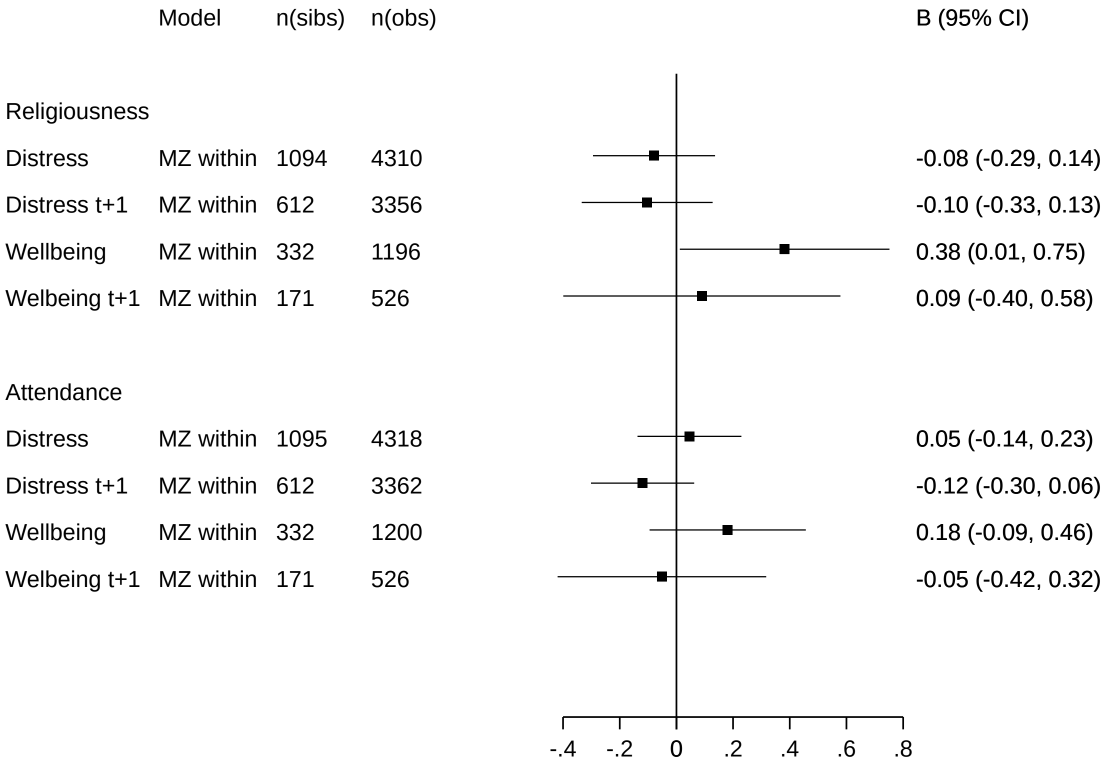

Web Figure 1. Summary estimates of random-effect meta-analyses for sibling analyses of religiosity predicting mental health in cross-sectional (t) and longitudinal (t+1) models, including only monozygotic twin pairs from AddHealth, MIDUS, and TwinLife (for psychological distress) and from MIDUS (for psychological wellbeing) cohort studies. Regression coefficients indicate the standardized mean difference in outcome between highest and lowest values of the predictor (i.e., not at all vs very religious for religiousness, and never vs. weekly for attendance). Overall model estimates are for analysis that does not apply fixed-effect estimation within sibling pairs, within model estimates apply fixed-effect estimation. n(sibs) = number of sibling pairs, n(obs) = total number of person-observations from multiple sibling pairs and across multiple measurement times.

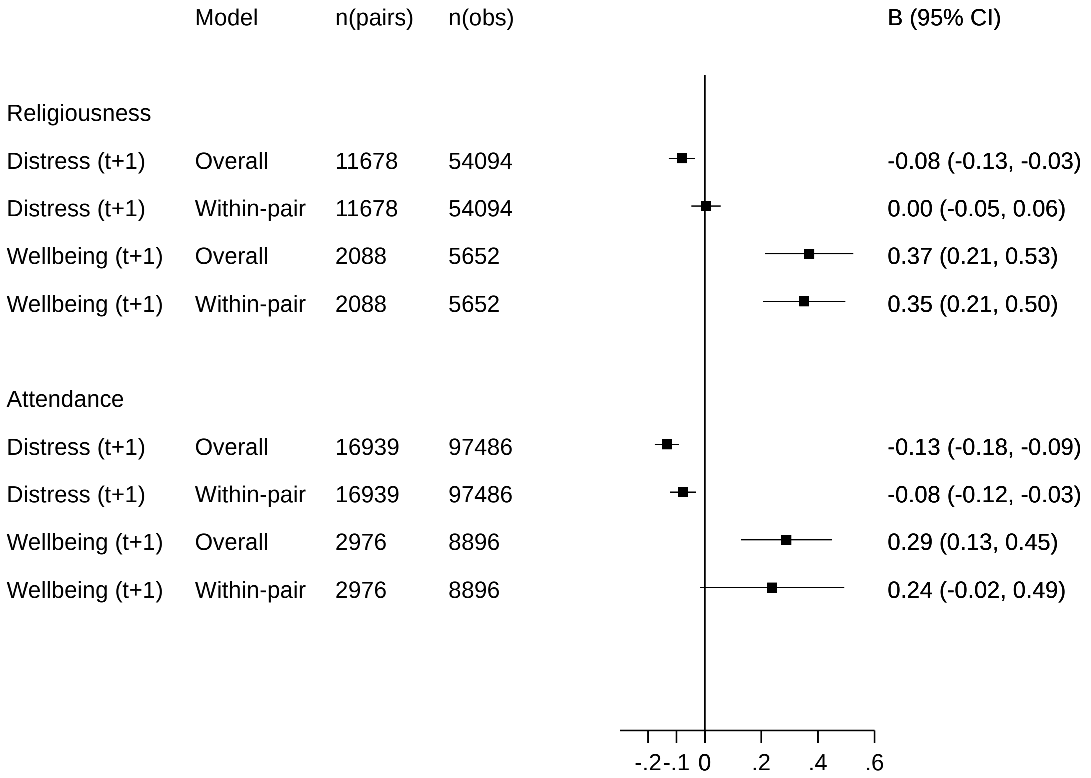

Web Figure 2. Summary estimates of random-effect meta-analyses for sibling analyses of religiosity predicting later mental health assessed in the following study wave T+1, when including only one sibling pair from the same family/household. Regression coefficients indicate the standardized mean difference in outcome between highest and lowest values of the predictor (i.e., not at all vs very religious for religiousness, and never vs. weekly for attendance). Overall model estimates are for analysis that does not apply fixed-effect estimation within sibling pairs, within model estimates apply fixed-effect estimation. n(pairs) = number of sibling pairs, n(obs) = total number of person-observations from multiple sibling pairs and across multiple measurement times.

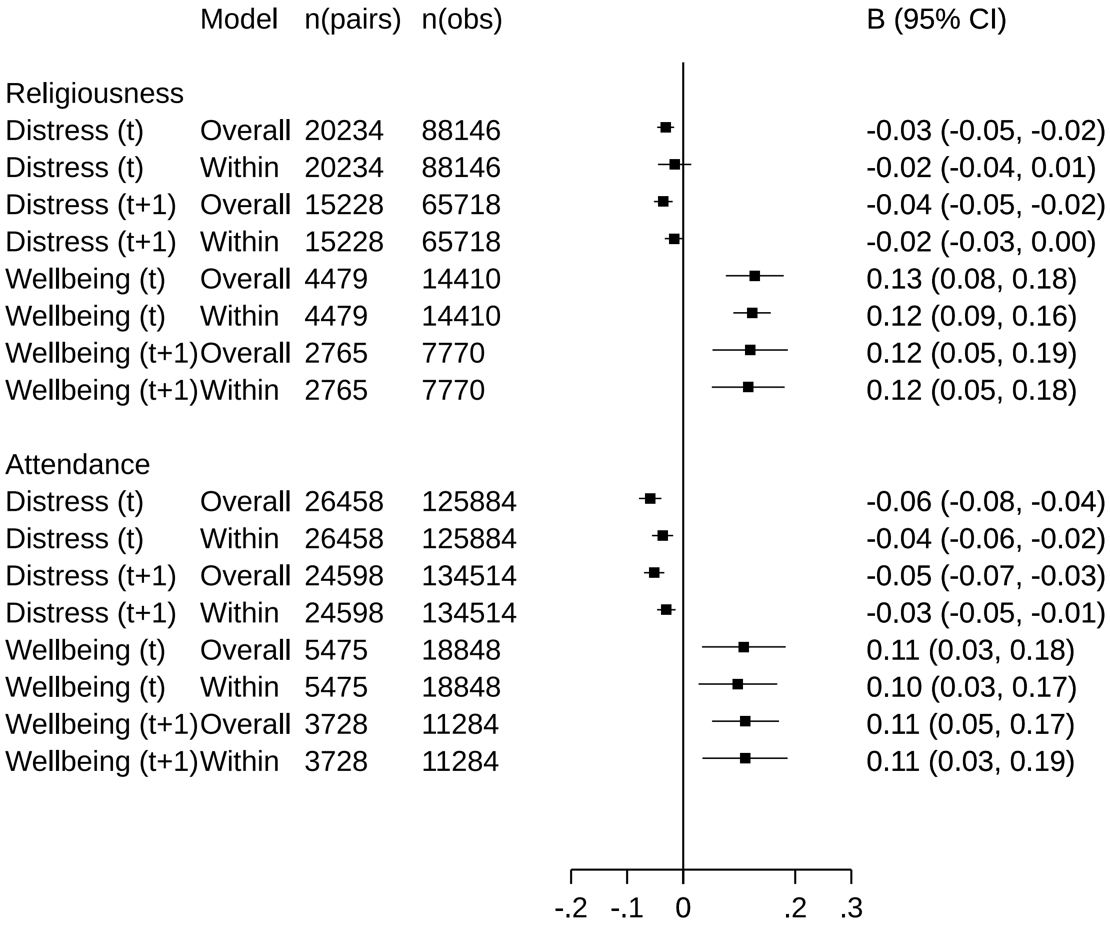

Web Figure 3. Summary estimates of random-effect meta-analyses for sibling analyses of religiosity and mental health in cross-sectional (t) and longitudinal (t+1) models expressed as standardized beta coefficients (SD=1 for exposure and outcome). Overall model estimates are for analysis that does not apply fixed-effect estimation within sibling pairs, within model estimates apply fixed-effect estimation. n(pairs) = number of sibling pairs, n(obs) = total number of person-observations from multiple sibling pairs and across multiple measurement times.

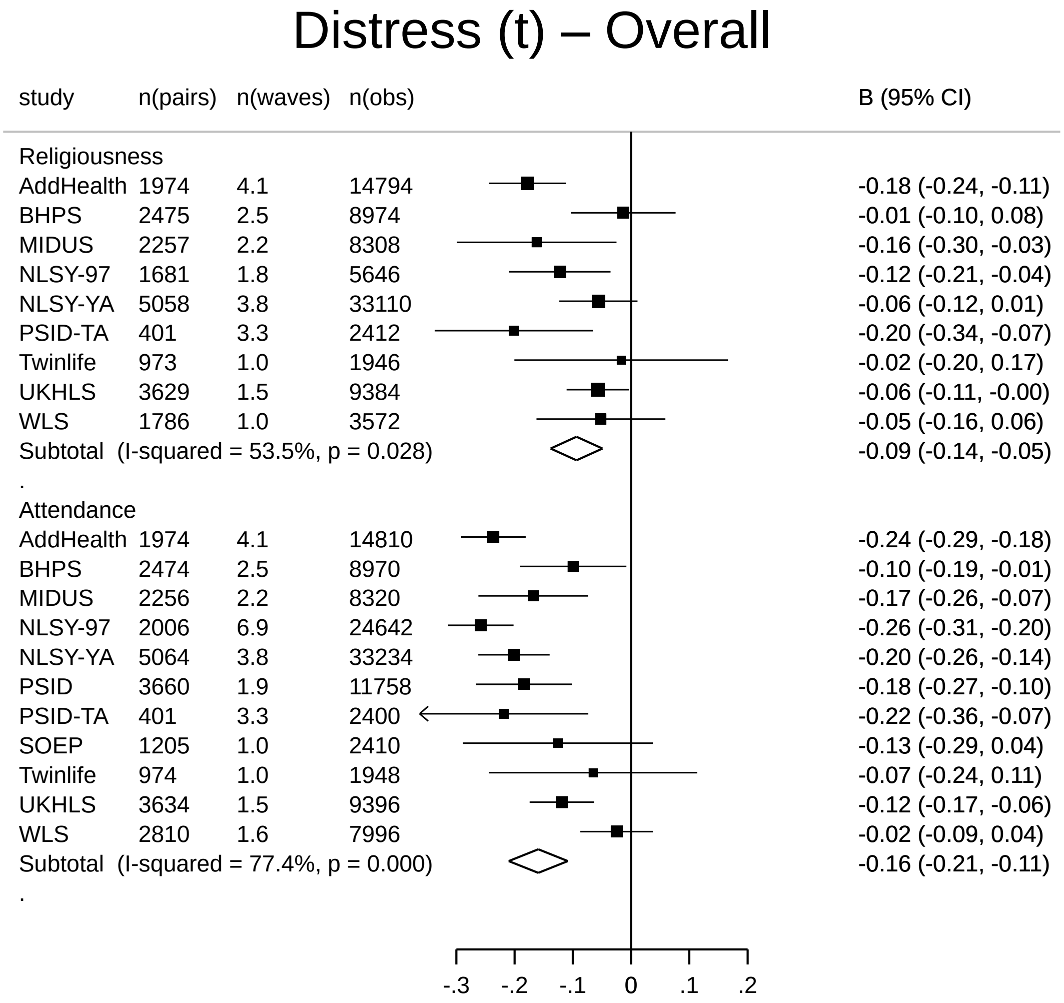

Web Figure 4. Cohort-specific cross-sectional associations between religiosity and psychological distress in the sibling samples.

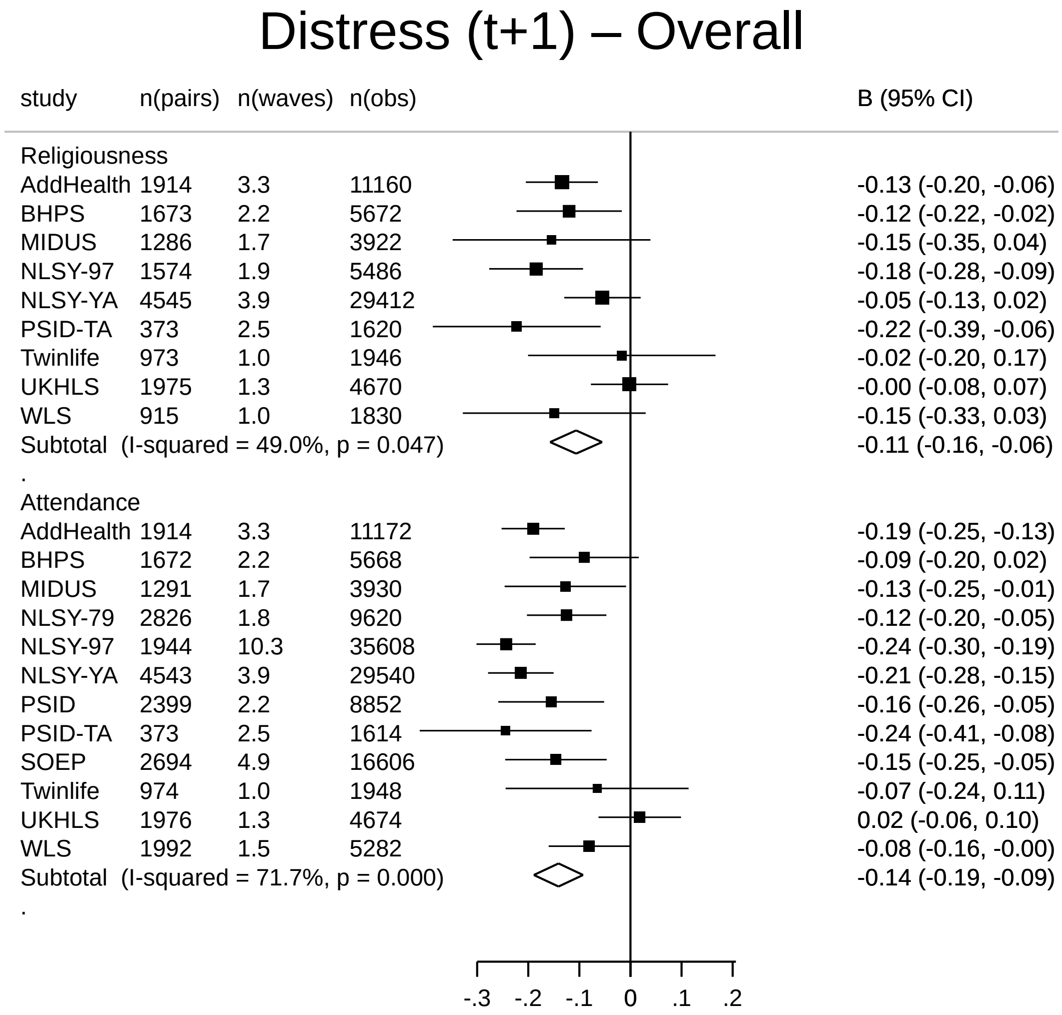

Web Figure 5. Cohort-specific longitudinal associations between religiosity and later psychological distress in the sibling samples.

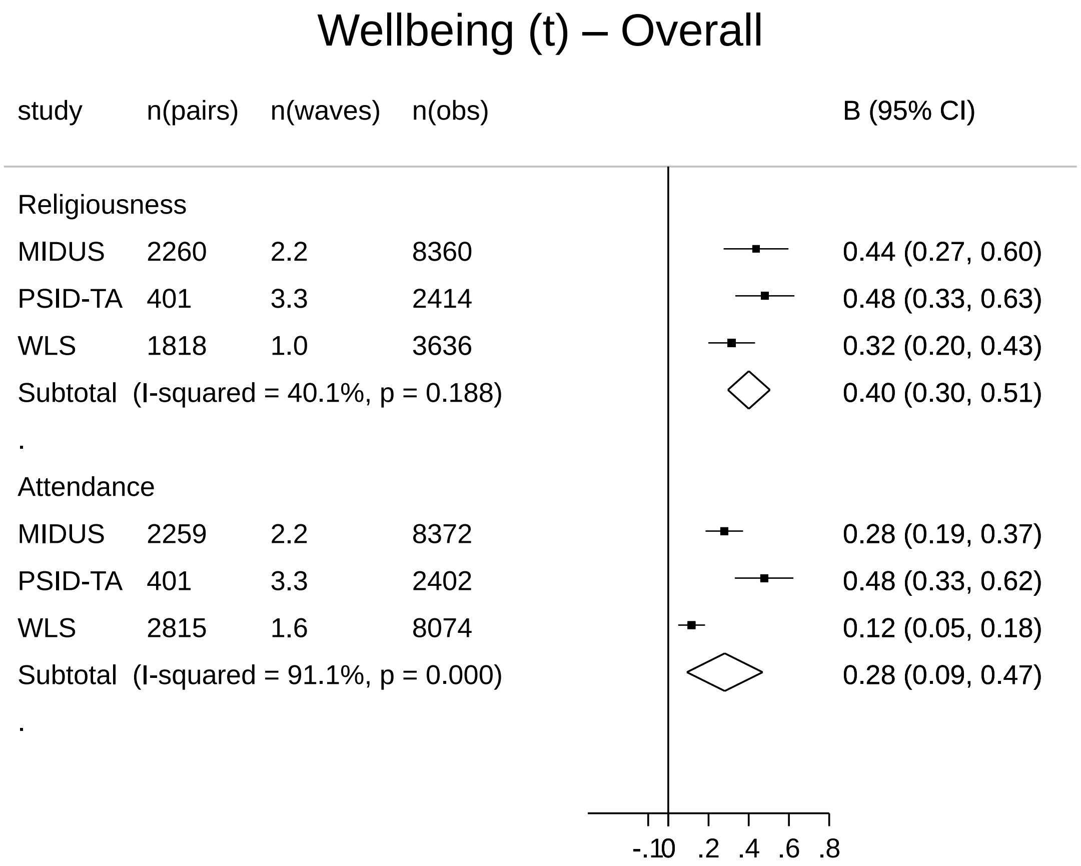

Web Figure 6. Cohort-specific cross-sectional associations between religiosity and psychological wellbeing in the sibling samples.

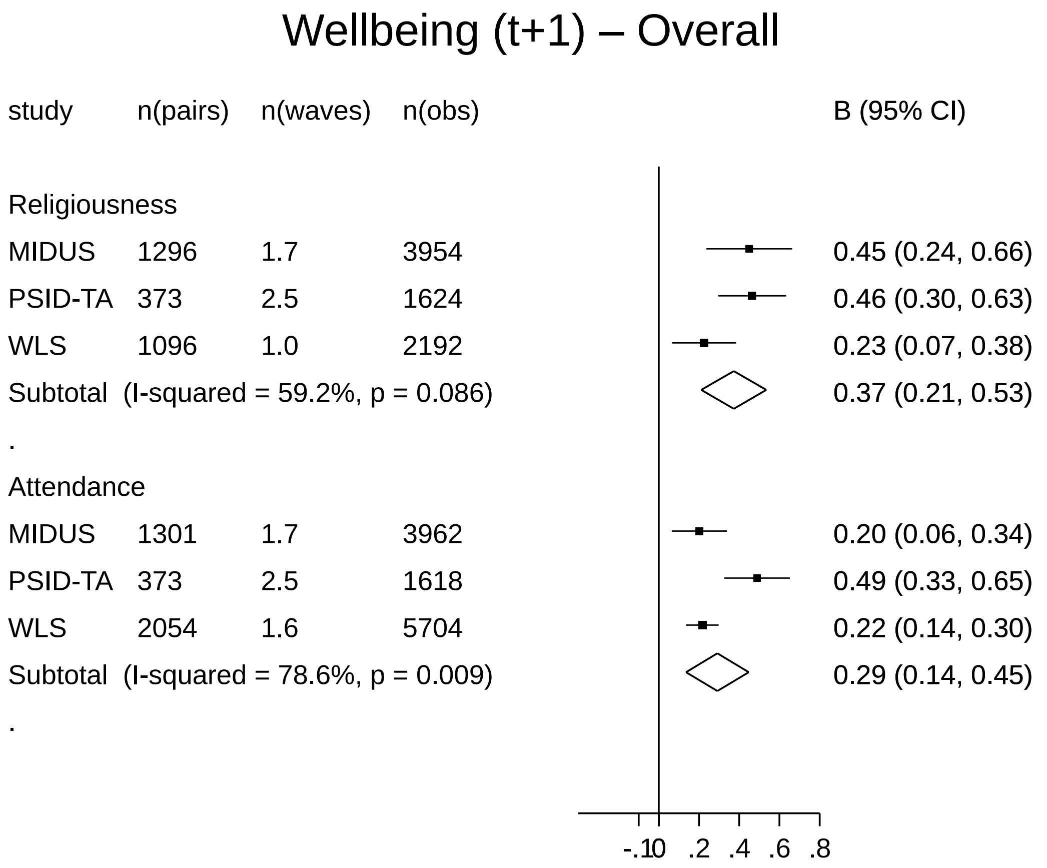

Web Figure 7. Cohort-specific longitudinal associations between religiosity and later psychological wellbeing in the sibling samples.

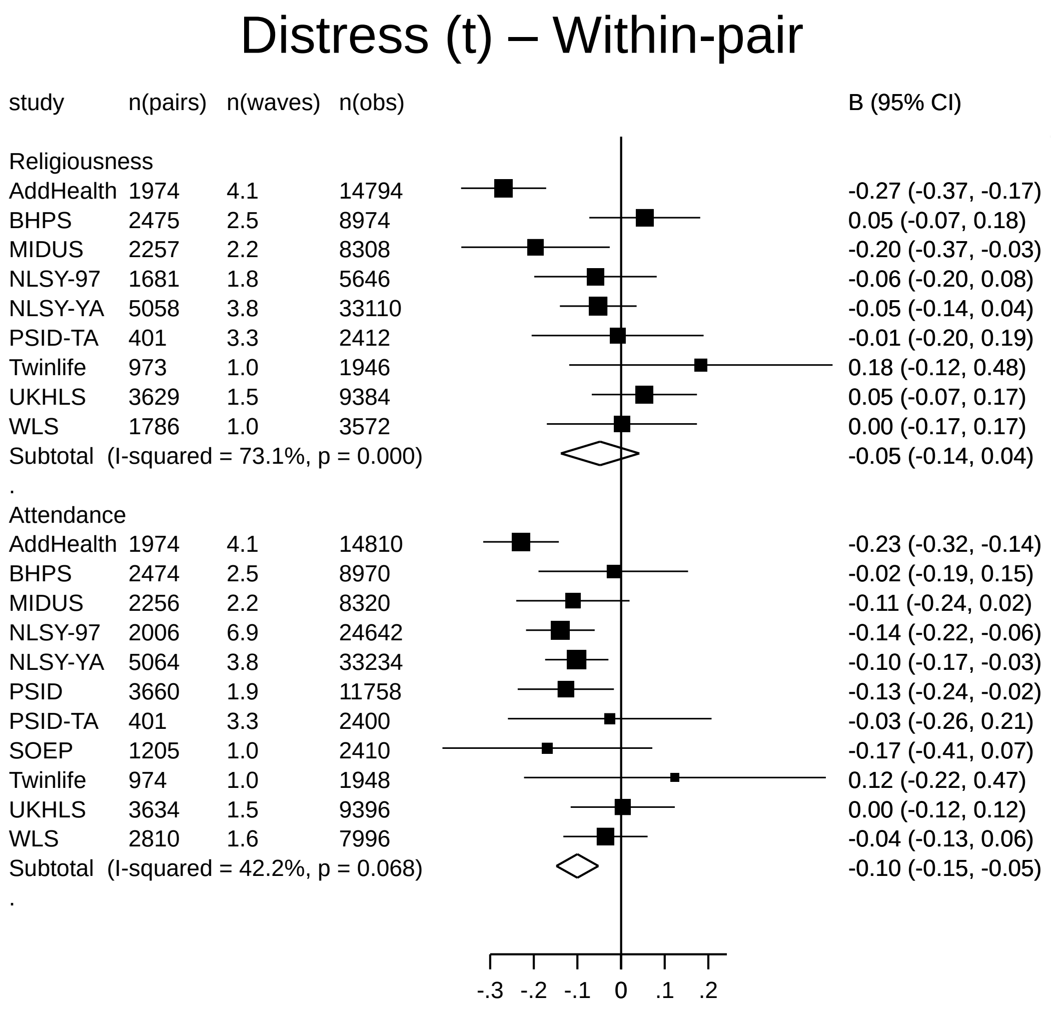

Web Figure 8. Cohort-specific cross-sectional associations between religiosity and psychological distress using within-sibling fixed-effect estimation in the sibling samples.

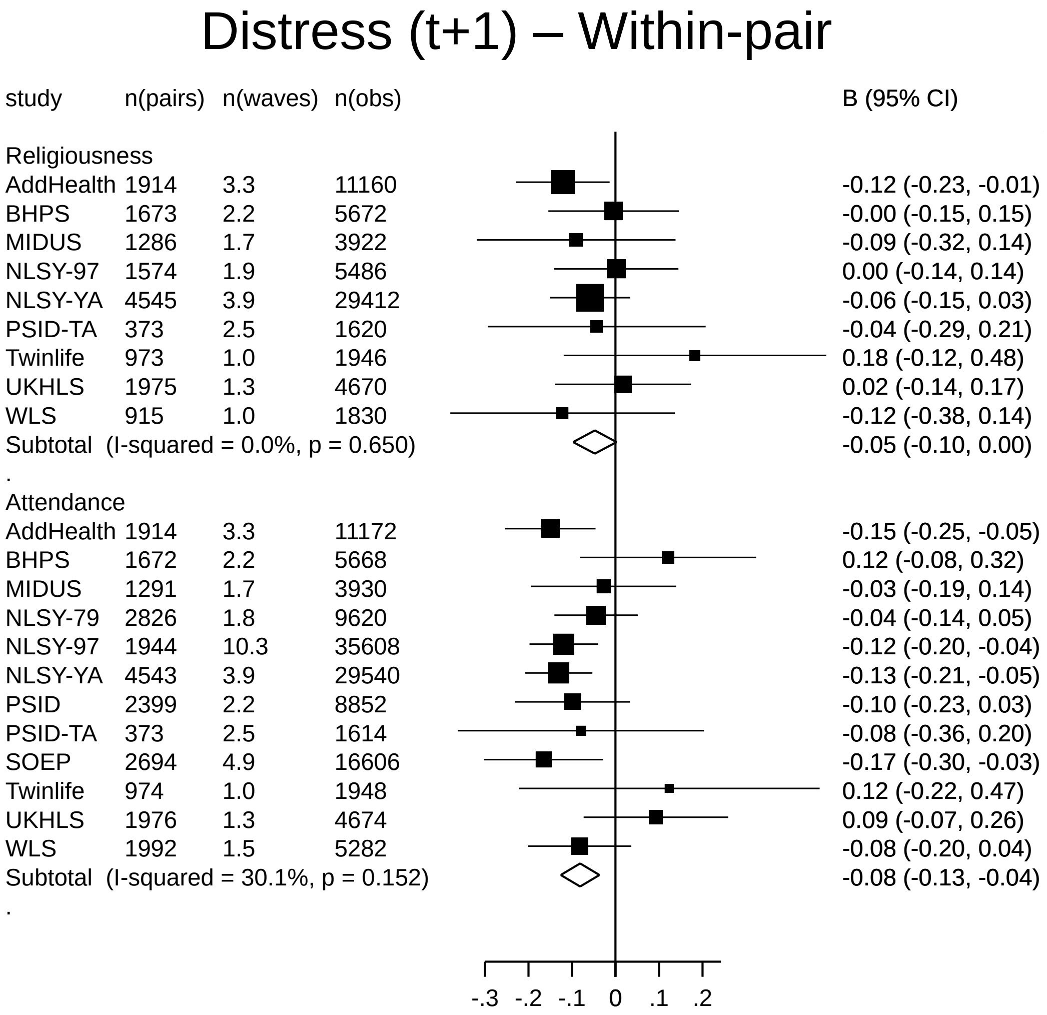

Web Figure 9. Cohort-specific longitudinal associations between religiosity and psychological distress using within-sibling fixed-effect estimation in the sibling samples.

Wellbeing (t) – Within-pair

study	n(pairs)	n(waves)	n(obs)	B (95% CI)
Religiousness				
MIDUS	2260	2.2	8360	0.48 (0.29, 0.67)
PSID-TA	401	3.3	2414	0.39 (0.15, 0.62)
WLS	1818	1.0	3636	0.37 (0.20, 0.54)
Subtotal (I-squared = 0.0%, p = 0.662)				0.41 (0.30, 0.52)
.				
Attendance				
MIDUS	2259	2.2	8372	0.25 (0.14, 0.36)
PSID-TA	401	3.3	2402	0.48 (0.26, 0.71)
WLS	2815	1.6	8074	0.10 (0.01, 0.20)
Subtotal (I-squared = 81.5%, p = 0.005)				0.25 (0.08, 0.43)
.				

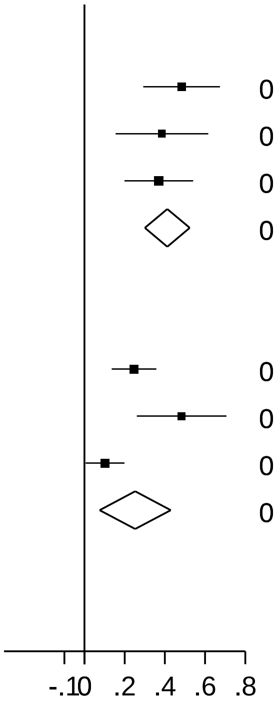

Web Figure 10. Cohort-specific cross-sectional associations between religiosity and psychological wellbeing using within-sibling fixed-effect estimation in the sibling samples.

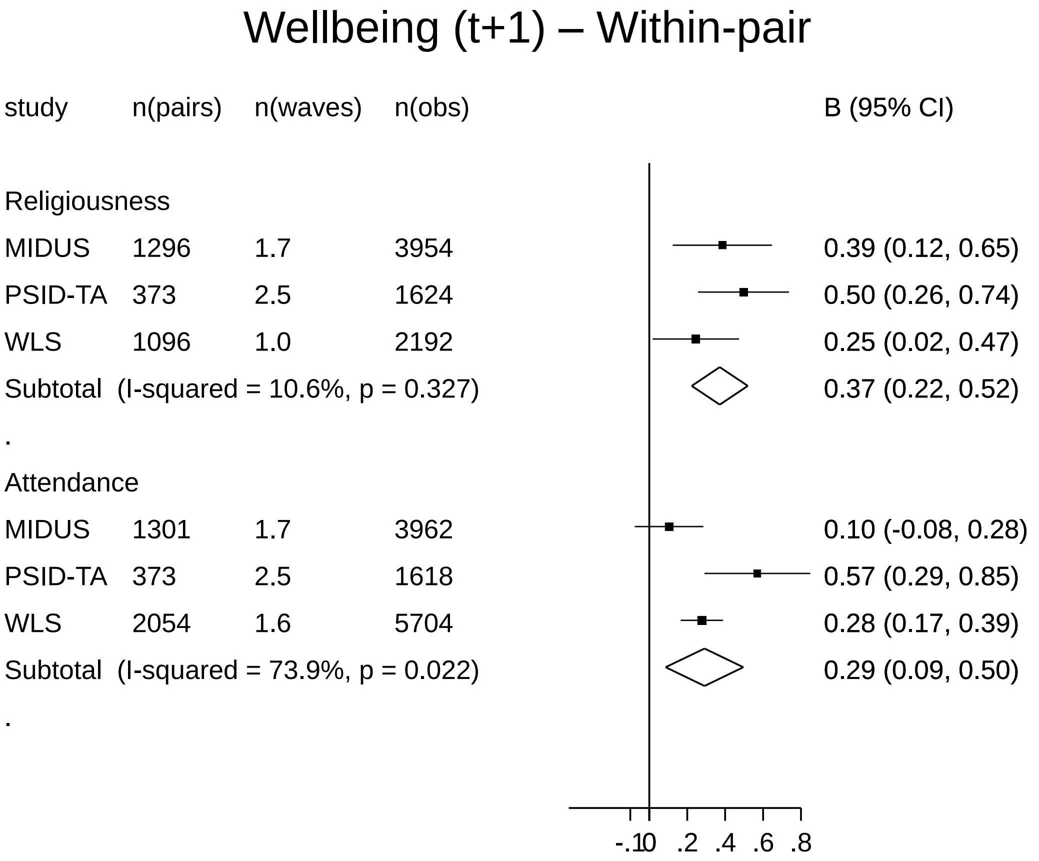

Web Figure 11. Cohort-specific longitudinal associations between religiosity and later psychological wellbeing using within-sibling fixed-effect estimation in the sibling samples.

Web Appendix 2

Within-individual analysis

I used repeated measurements of religiosity and mental health to assess within-individual associations that can adjust for any confounding factors that remain stable within individuals across measurement times, as the individual acts as the reference for him/herself, rather than being compared with other individual. Thus, within-individual analysis can assess whether the person has lower psychological distress in times when the person attends religious activities compared to other times when the person does not attend religious activities.

Random-intercept, multilevel regression was used to estimate the associations, and fixed-effect estimator (also known as group-mean centering or within-individual analysis) was used to test whether differences in religiosity between siblings were related to the siblings' differences in mental health, and whether differences in religiosity within the same individual over repeated measurement times were related to differences in mental health. In all analyses, each participant could contribute multiple person-observations if the participant had two or more repeated measurements of both religiosity and mental health.

Web Table 9 shows the transition matrices for religiousness and religious attendance over time, that is, the probabilities of participants moving across religiosity categories across measurement times T and T+1. **Web Table 10** cross-tabulates the minimum and maximum values of the variables within individuals to show how many individuals there were in each group of maximum range variance. The intraclass correlations and transition matrices suggested moderately high stability in religious attendance and religiousness, but there were still >14,000 participants who reported attending religious services both never and weekly at least in one of the repeated study waves (n>5000 for religiousness). This provided sufficient variation in religiosity for the fixed-effect regressions.

Web Figures 12 and **13** show the within-individual associations. When using the fixed-effect estimation that only considered the individual's variation around the individual's own mean over time, most of the associations attenuated substantially and none of the estimates remained statistically significant or within the confidence intervals to the overall estimates. The dose–response associations were also attenuated to null in the within-individual analysis (**Web Figure 14**), as were the associations of reverse temporal order (**Web Figure 15**). **Web Figures 16** to **24** show the cohort-specific results of the within-individual models.

In sum, the within-individual associations suggested that people's psychological distress and psychological wellbeing are unlikely to change when people's level of religiousness or religious attendance changes over time. These findings need to be considered within the potential limitations of the within-individual analysis, which assumes no time-varying confounding and no reverse causation, both of which are plausible sources of bias in the present case even if the magnitude of this bias is not known.

Web Table 9. Intraclass correlations of the variables in the sibling analysis and within-individual longitudinal analysis

Within individuals over time				
	Religious attendance	Religiousness	Psychological distress	Psychological wellbeing
AddHealth	0.40	0.42	0.34	–
BHPS	0.77	0.66	0.41	–
HRS	0.75	0.75	0.43	0.44
MIDUS	0.52	0.67	0.55	0.64
NLSY79	0.30	–	–	–
NLSY97	0.53	0.69	0.39	–
NLSY-YA	0.47	0.56	0.34	–
PSID	0.62	–	0.51	–
PSID-TA	0.61	0.65	0.49	0.46
SOEP	0.75	–	.	–
TWINLIFE	–	–	–	–
UKHLS	0.79	0.73	0.46	–
WLS	0.70	–	0.48	0.70

Note: Values are intraclass correlations of repeated measurements within individuals in a pooled sample of all the cohort studies, indicating the proportion of variance in the variable accounted for by average individual differences across measurement times.

Web Table 10. Transition probabilities of individuals moving between categories of religiosity between study waves (t) and (t+1)

	Never (t+1)	Few times a year (t+1)	Monthy (t+1)	Weekly (t+1)
Never (t)	80.3	14.6	2.9	2.3
Few times a year (t)	28.5	53.2	12.2	6.1
Monthy (t)	10.9	29.1	39.9	19.9
Weekly (t)	4.8	8.8	14.9	71.1

	Not religious (t+1)	Somewhat religious (t+1)	Religious (t+1)	Very religious (t+1)
Not religious (t)	71.0	14.2	11.2	3.5
Somewhat religious (t)	30.3	40.7	24.7	4.3
Religious (t)	10.2	16.3	53.8	19.6
Very religious (t)	2.3	2.8	18.3	76.6

Note: Values are probabilities (%) of participants moving between categories of religious attendance and religiousness between two consecutive study waves. For example, 80.3% of participants who reported not ever going to religious services reported not going to religious services also in the next study wave, while 2.3% of them reported attending religious services weekly in the next study wave.

Web Table 11. Number of participants by the individuals' minimum and maximum values of religiosity throughout the follow-up period

	Never (max)	Few times a year (max)	Monthy (max)	Weekly (max)	Total
Never (min)	83325	30368	11955	14565	140213
Few times a year (min)	0	21252	9395	13264	43911
Monthy (min)	0	0	10681	11831	22512
Weekly (min)	0	0	0	33588	33588
Total	83325	51620	32031	73248	240224

	Not religious (max)	Somewhat religious (max)	Religious (max)	Very religious (max)	Total
Not religious (min)	38484	11732	11445	5647	67308
Somewhat religious (min)	0	15890	9892	5579	31361
Religious (min)	0	0	21011	17685	38696
Very religious (min)	0	0	0	31145	31145
Total	38484	27622	42348	60056	168510

Note: Values are numbers of participants categorized based on the lowest (min) and highest (max) values of religious attendance and religiousness throughout the follow-up time. For example, 83,325 participants reported never attending religious services in all the study waves, while 14,565 participants reported attending never in at least one study wave and attending weekly in at least another study wave throughout the follow-up period.

	Model	n(persons)	n(obs)	B (95% CI)
Religiousness				
Distress (t+1)	Overall	117326	291674	-0.05 (-0.10, 0.00)
Distress (t+1)	Within	117326	291674	-0.00 (-0.04, 0.03)
Wellbeing (t+1)	Overall	21852	43225	0.18 (0.13, 0.23)
Wellbeing (t+1)	Within	21852	43225	-0.03 (-0.09, 0.02)
Attendance				
Distress (t+1)	Overall	191400	636102	-0.10 (-0.11, -0.08)
Distress (t+1)	Within	191400	636102	-0.02 (-0.04, 0.00)
Wellbeing (t+1)	Overall	32203	61831	0.19 (0.15, 0.23)
Wellbeing (t+1)	Within	32203	61831	0.01 (-0.08, 0.09)

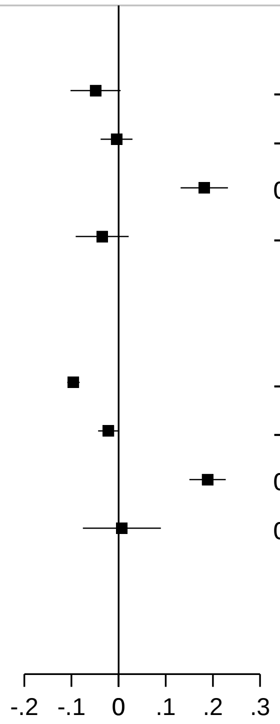

Web Figure 12. Summary estimates of random-effect meta-analyses of within-individual analyses in which religiosity (religiousness and religious attendance, separate models) at wave T was used to predict subsequent distress or wellbeing in the following study wave T+1. Regression coefficients indicate the standardized mean difference in outcome between highest and lowest values of the predictor (i.e., not at all vs very religious for religiousness, and never vs. weekly for attendance). “Overall” models apply ordinary regression, “within” models apply fixed-effect estimation within repeated measurements of individuals. n(persons) = number of unique participants, n(obs) = total number of person-observations across multiple measurement times.

	Model	n(persons)	n(obs)	B (95% CI)
Religiousness				
Distress (t)	Overall	156543	411112	-0.05 (-0.09, 0.00)
Distress (t)	Within	127190	381759	-0.02 (-0.06, 0.02)
Distress (t+1)	Overall	117326	291674	-0.05 (-0.10, 0.00)
Distress (t+1)	Within	117326	291674	-0.00 (-0.04, 0.03)
Wellbeing (t)	Overall	25690	49304	0.26 (0.15, 0.37)
Wellbeing (t)	Within	25690	49304	0.13 (0.04, 0.22)
Wellbeing (t+1)	Overall	21852	43225	0.18 (0.13, 0.23)
Wellbeing (t+1)	Within	21852	43225	-0.03 (-0.09, 0.02)
Attendance				
Distress (t)	Overall	184498	570221	-0.05 (-0.06, -0.04)
Distress (t)	Within	154061	539784	-0.03 (-0.04, -0.02)
Distress (t+1)	Overall	191400	636102	-0.04 (-0.04, -0.03)
Distress (t+1)	Within	191400	636102	-0.01 (-0.02, 0.00)
Wellbeing (t)	Overall	37582	75432	0.08 (0.05, 0.11)
Wellbeing (t)	Within	37582	75432	0.03 (0.01, 0.06)
Wellbeing (t+1)	Overall	32203	61831	0.07 (0.06, 0.09)
Wellbeing (t+1)	Within	32203	61831	0.00 (-0.03, 0.04)

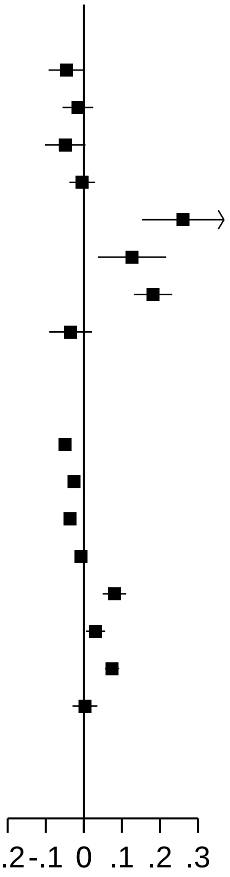

Web Figure 13. Summary estimates of random-effect meta-analyses for within-individual analyses of religiosity and mental health in cross-sectional (t) and longitudinal (t+1) models expressed as standardized beta coefficients (SD=1 for exposure and outcome). Overall model estimates are for analysis that does not apply fixed-effect estimation within the repeated measurements of individuals, within estimates apply fixed-effect estimation. n(persons) = number of unique participants, n(obs) = total number of person-observations across multiple measurement times.

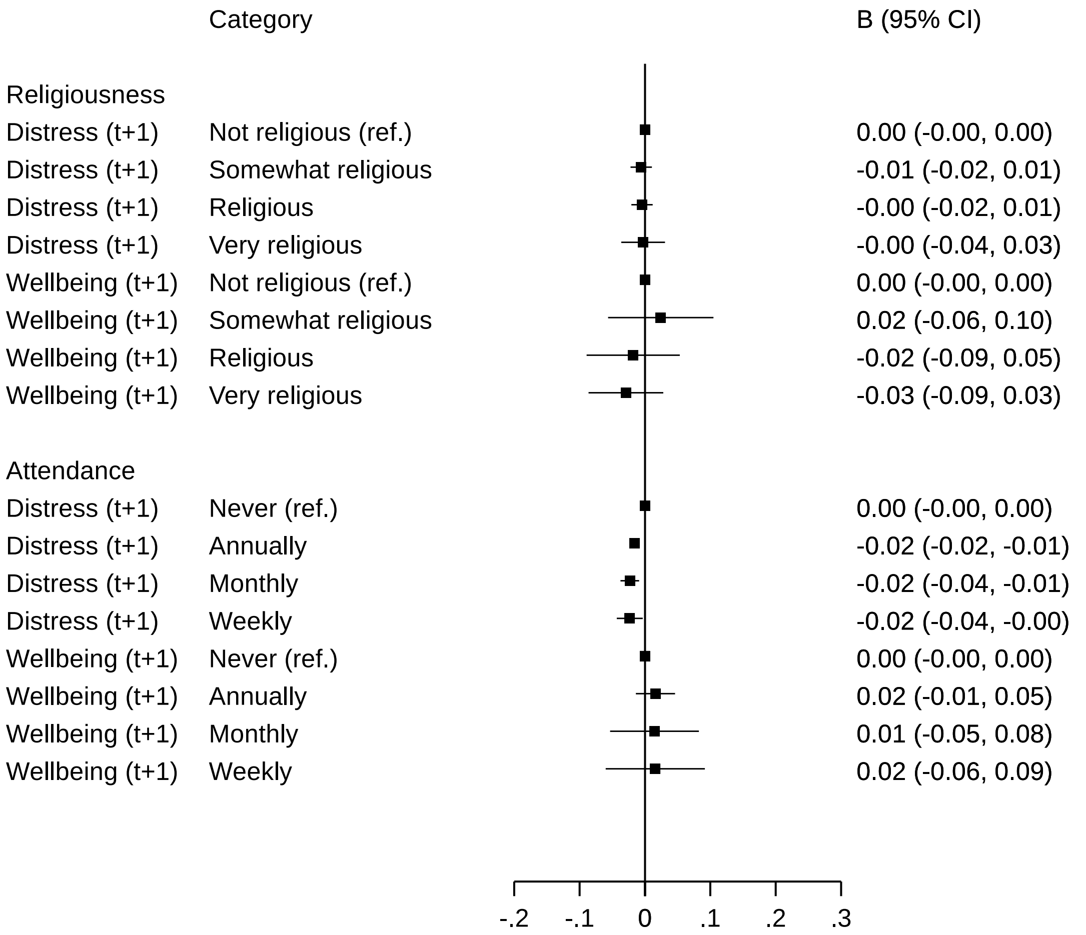

Web Figure 14. Summary estimates of random-effect meta-analyses for dose–response analyses of religiosity (religiousness and religious attendance, separate models) and subsequent mental health assessed in the following study wave T+1 when using the fixed-effect regression estimation. Regression coefficients indicate the standardized mean difference in outcome compared to the reference group.

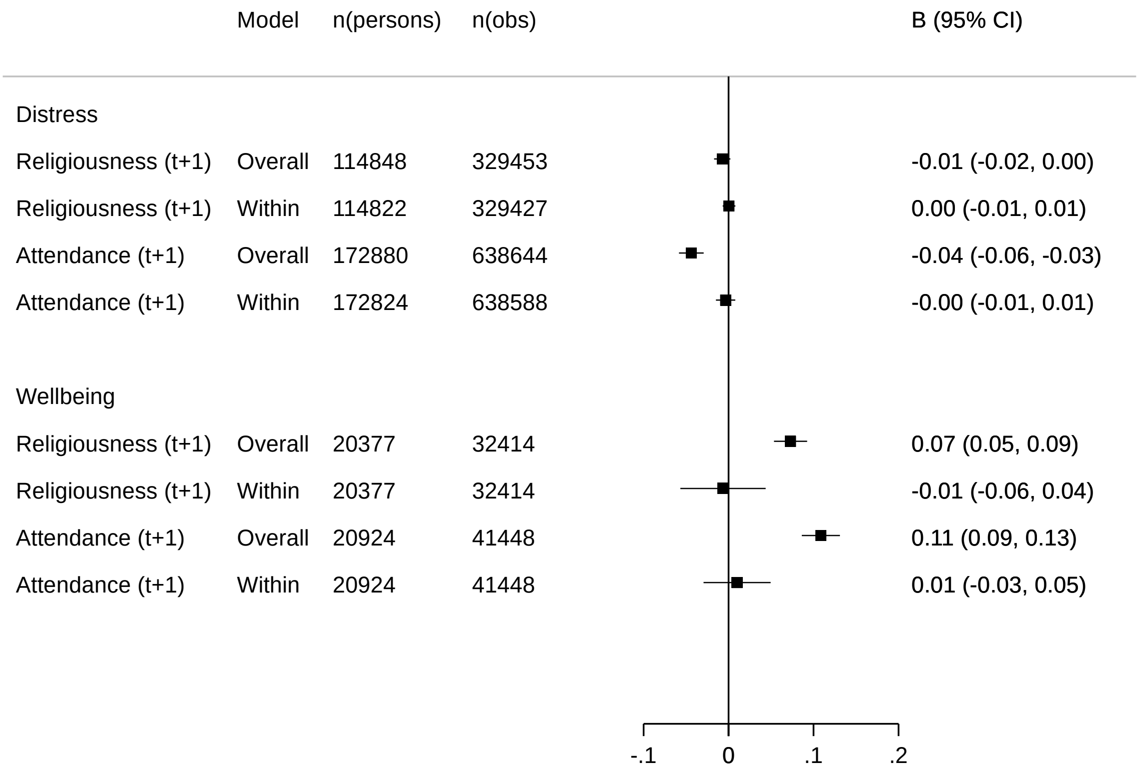

Web Figure 15. Summary estimates of random-effect meta-analyses of reverse temporal order in the within-individual analysis, that is, mental health predicting later religiosity assessed in the following study wave T+1. Regression coefficients indicate the standardized mean difference in outcome between highest and lowest values of the predictor (i.e., not at all vs very religious for religiousness, and never vs. weekly for attendance). Overall model estimates are for analysis that does not apply fixed-effect estimation within the repeated measurements of individuals, within estimates apply fixed-effect estimation. n(persons) = number of unique participants, n(obs) = total number of person-observations across multiple measurement times.

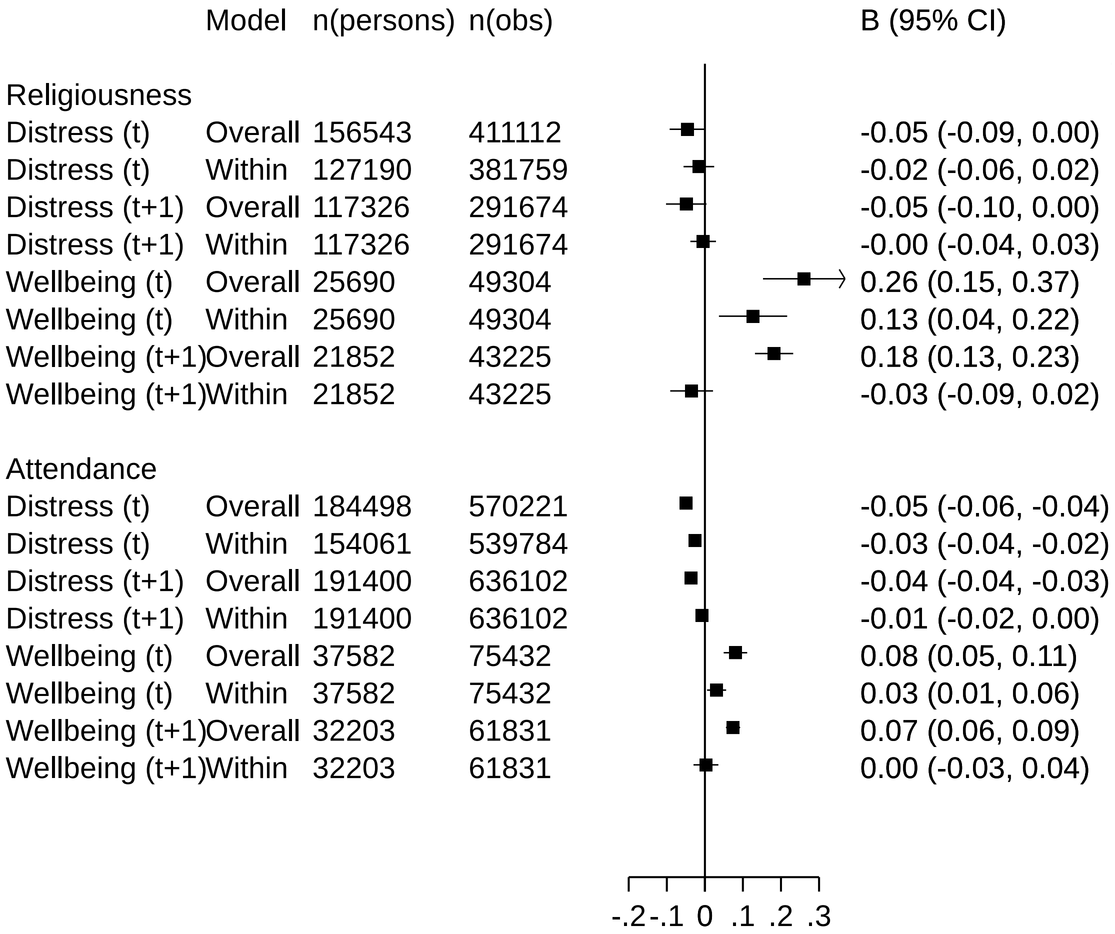

Web Figure 16. Summary estimates of random-effect meta-analyses for within-individual analyses of religiosity and mental health in cross-sectional (t) and longitudinal (t+1) models expressed as standardized beta coefficients (SD=1 for exposure and outcome). Overall model estimates are for analysis that does not apply fixed-effect estimation within the repeated measurements of individuals, within estimates apply fixed-effect estimation. n(persons) = number of unique participants, n(obs) = total number of person-observations across multiple measurement times.

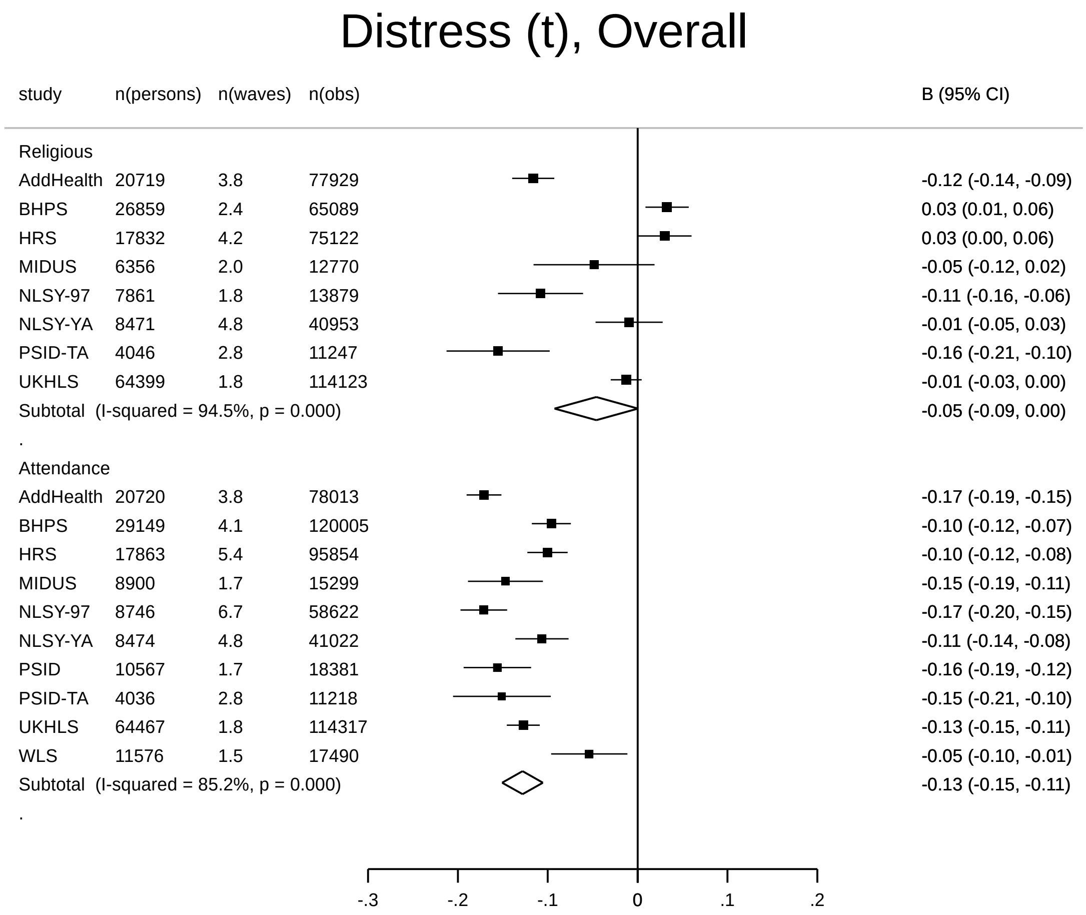

Web Figure 17. Cohort-specific cross-sectional associations between religiosity and psychological distress in the full samples.

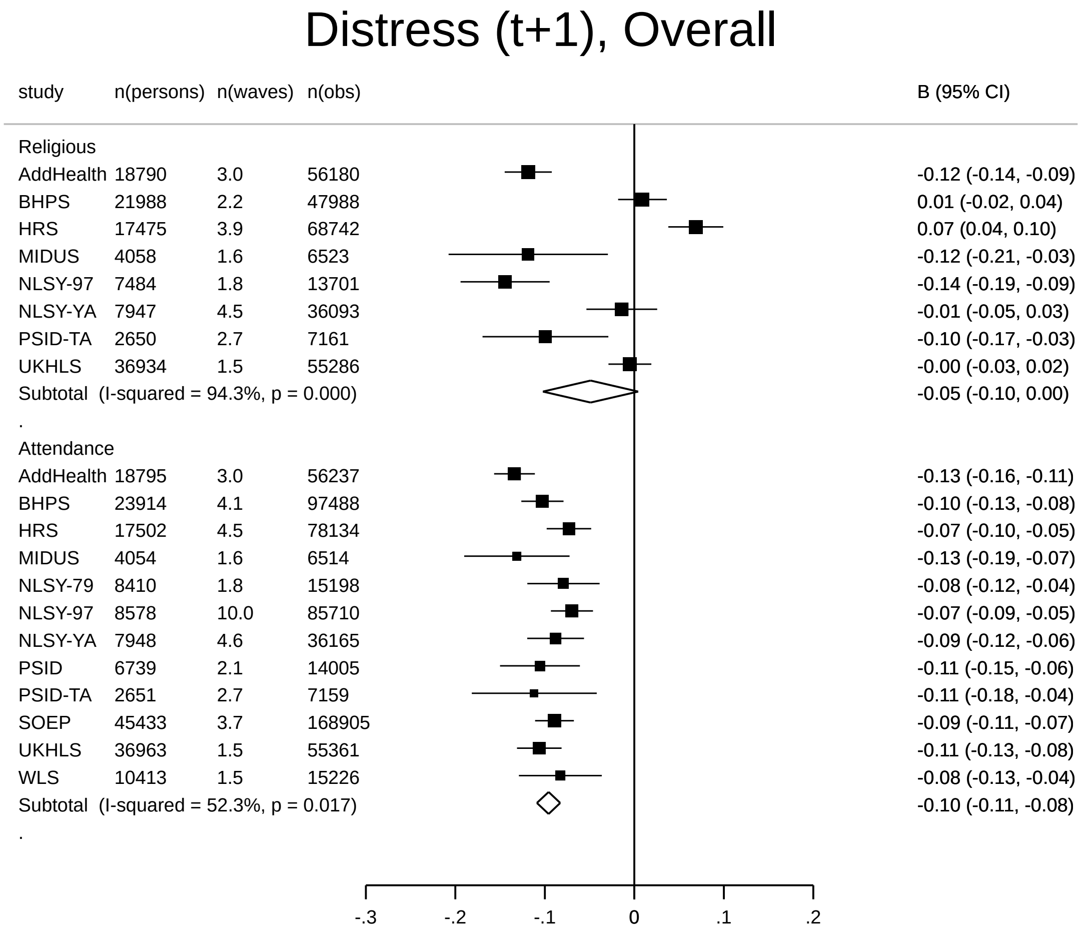

Web Figure 18. Cohort-specific longitudinal associations between religiosity and psychological distress in the full samples.

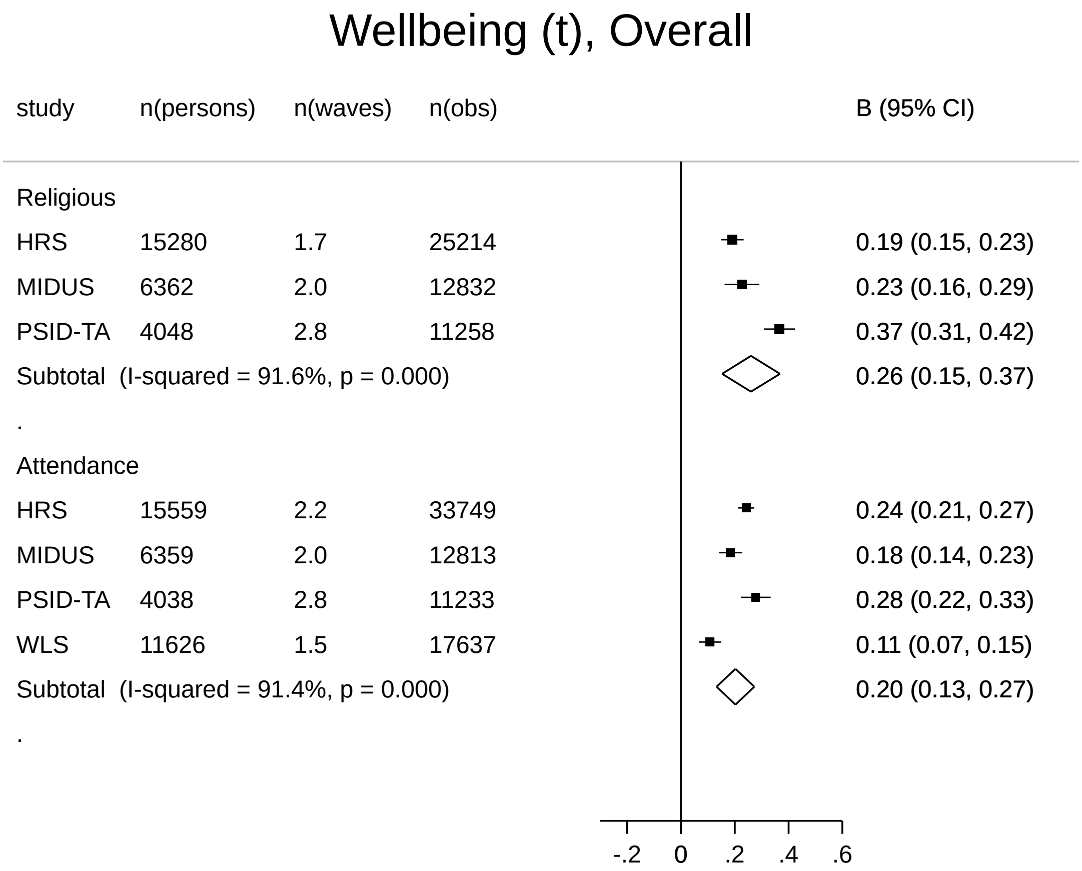

Web Figure 19. Cohort-specific cross-sectional associations between religiosity and psychological wellbeing in the full samples.

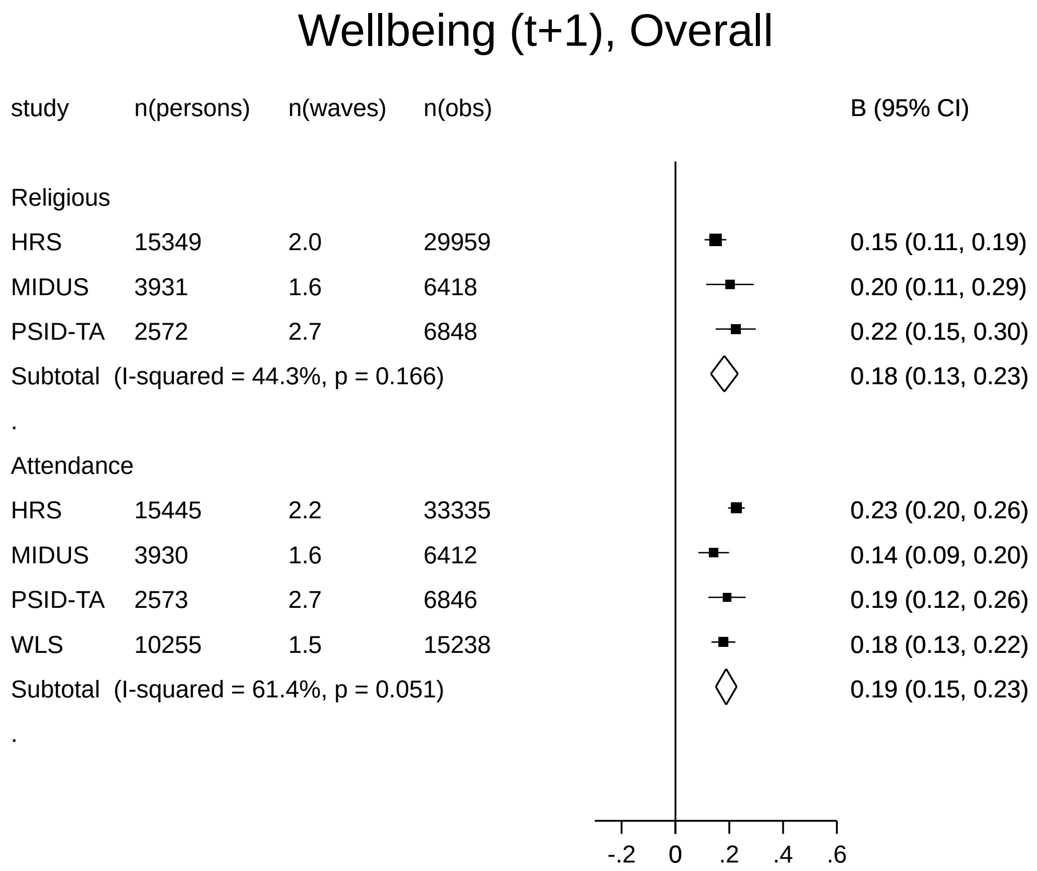

Web Figure 20. Cohort-specific longitudinal associations between religiosity and psychological wellbeing in the full samples.

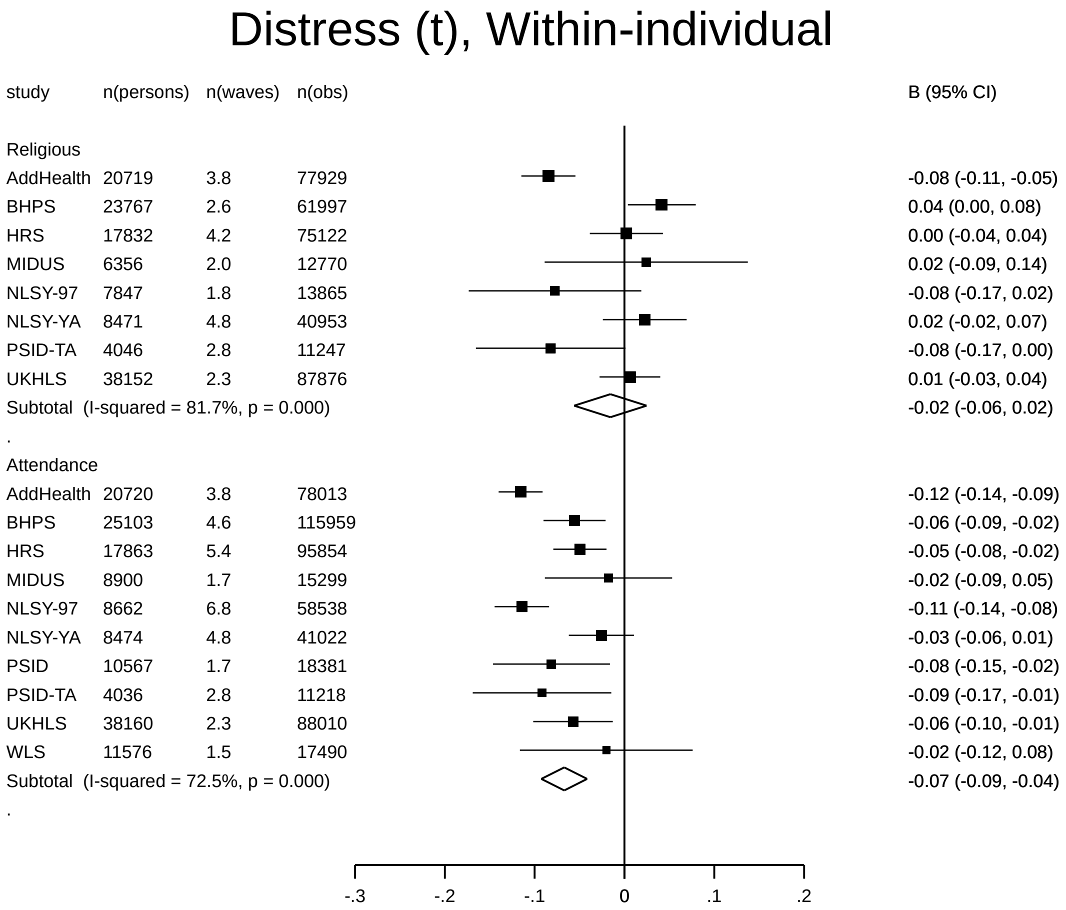

Web Figure 21. Cohort-specific cross-sectional associations between religiosity and psychological distress in the full samples using fixed-effect estimation of within-individual associations.

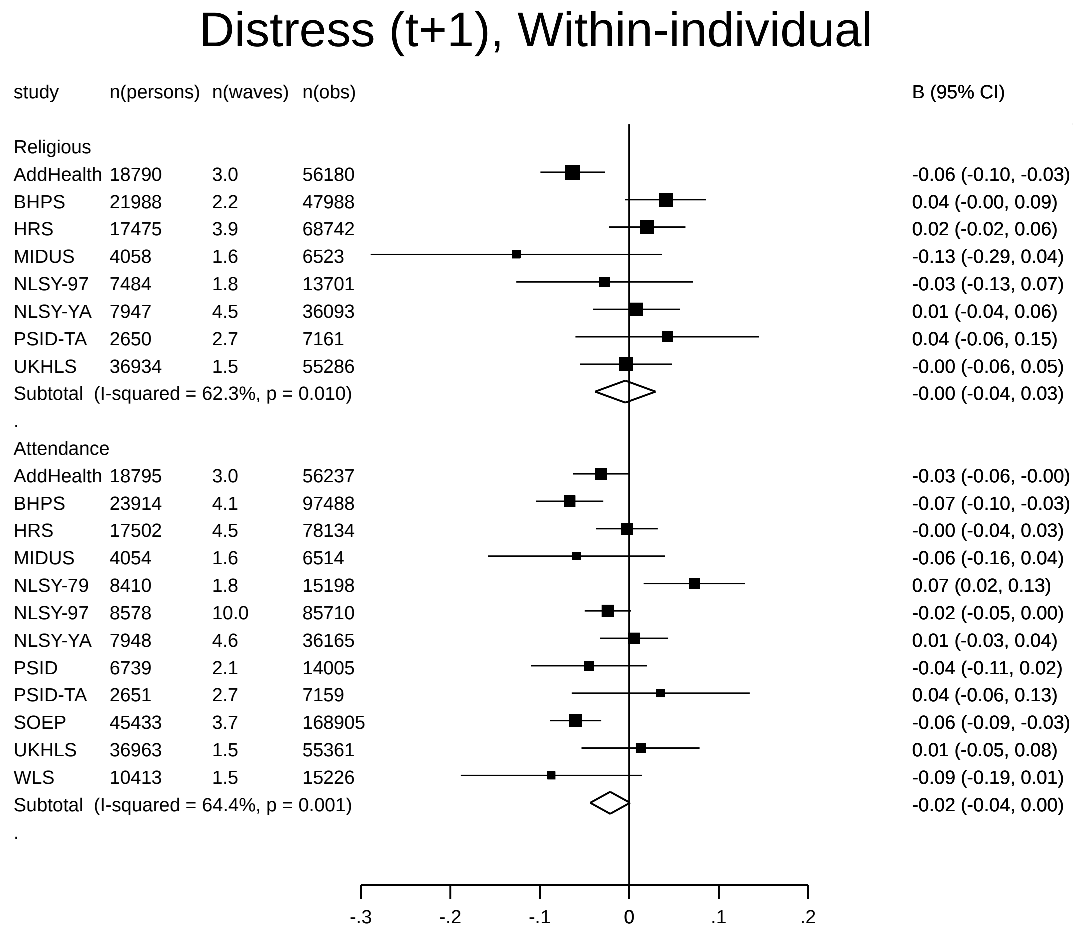

Web Figure 22. Cohort-specific longitudinal associations between religiosity and psychological distress in the full samples using fixed-effect estimation of within-individual associations.

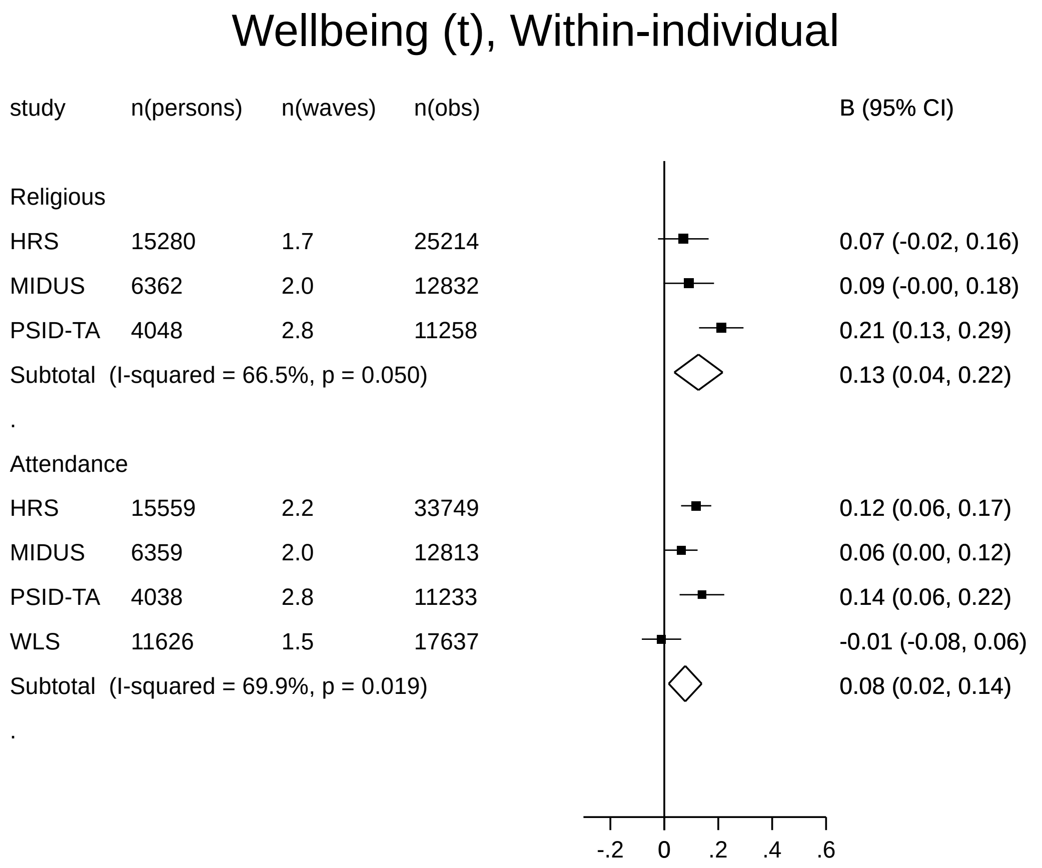

Web Figure 23. Cohort-specific cross-sectional associations between religiosity and psychological wellbeing in the full samples using fixed-effect estimation of within-individual associations.

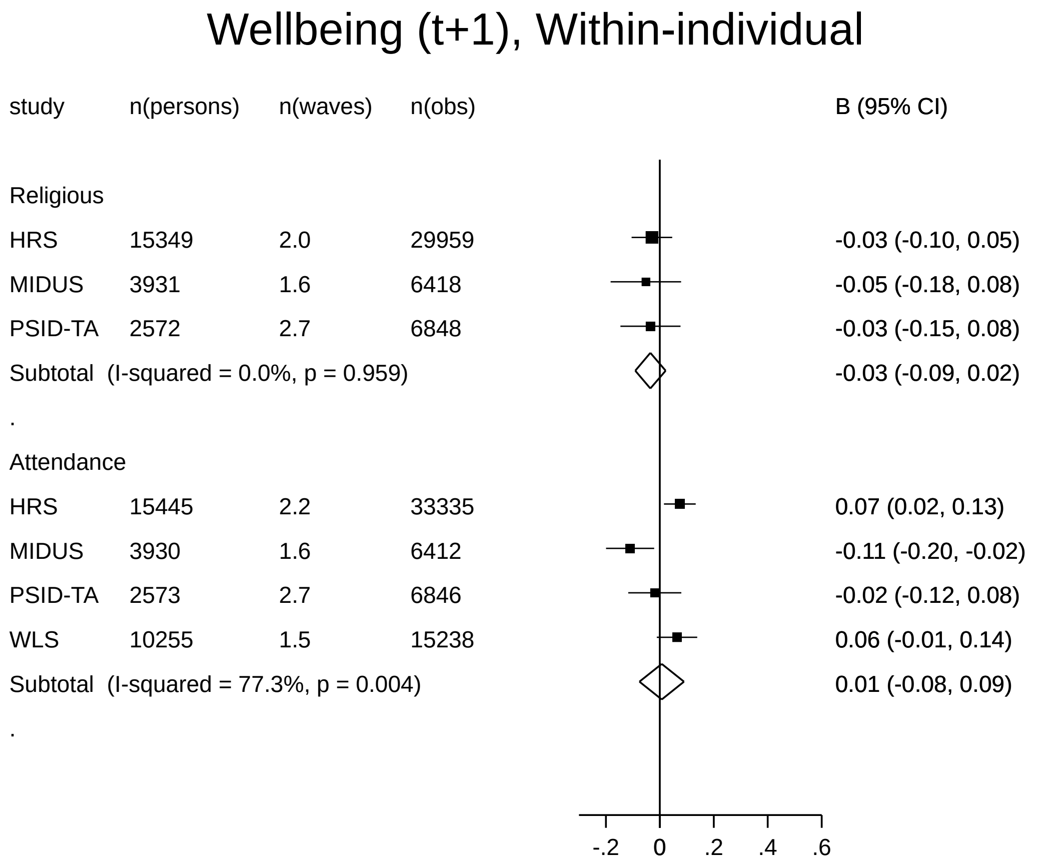

Web Figure 24. Cohort-specific longitudinal associations between religiosity and psychological wellbeing in the full samples using fixed-effect estimation of within-individual associations.
